# Moral values, social ideologies and threat-based cognition: Implications for intergroup relations

**DOI:** 10.3389/fpsyg.2022.869121

**Published:** 2022-10-06

**Authors:** David S. M. Morris, Brandon D. Stewart

**Affiliations:** ^1^School of Psychology, University of Birmingham, Birmingham, United Kingdom; ^2^Department of Psychology, University of Southern Maine, Portland, ME, United States

**Keywords:** moral foundations theory, ideology, threat, intergroup relations, authoritarianism, social dominance orientation

## Abstract

Moral foundations theory (MFT) has provided an account of the moral values that underscore different cultural and political ideologies, and these moral values of harm, fairness, loyalty, authority, and purity can help to explain differences in political and cultural ideologies; however, the extent to which moral foundations relate to strong social ideologies, intergroup processes and threat perceptions is still underdeveloped. To explore this relationship, we conducted two studies. In Study 1 (*N* = 157), we considered how the moral foundations predicted strong social ideologies such as authoritarianism (RWA) and social dominance orientation (SDO) as well as attitudes toward immigrants. Here, we demonstrated that more endorsement of individualizing moral foundations (average of harm and fairness) was related to less negative intergroup attitudes, which was mediated by SDO, and that more endorsement of binding moral foundations (the average of loyalty, authority, and purity) was related to more negative attitudes, which was mediated by RWA. Crucially, further analyses also suggested the importance of threat perceptions as an underlying explanatory variable. Study 2 (*N* = 388) replicated these findings and extended them by measuring attitudes toward a different group reflecting an ethnic minority in the United States, and by testing the ordering of variables while also replicating and confirming the threat effects. These studies have important implications for using MFT to understand strong ideologies, intergroup relations, and threat perceptions.

## Introduction

Moral foundations theory (MFT) has presented a compelling way to understand differences in social-political opinions and cultural attitudes. It has demonstrated that ideologies may be underscored by differences in moral values where each side believes that they are right and use different moral values to justify their attitudes, opinions, and beliefs (Haidt and Joseph, 2004; Graham et al., 2009; Haidt, 2012). MFT suggests that humans have evolved five core moral values that underlie our beliefs and attitudes and are expressed differentially between groups and cultures. These five moral values have further been used to explain differences observed between different political ideologies (Haidt, 2007, 2012; Graham et al., 2009). MFT identifies different moral values with important implications for social cognition and social judgments. For example the moral foundation of *harm* reflects concerns around reduced harm toward humans and animals, and the foundation of *fairness* reflects an emphasis on equality toward others. These are collectively named the “*individualizing*” moral foundations because they are said to relate to concerns based on the moral rights of individual members of society (Graham et al., 2009; Haidt, 2012).

Three additional moral values have been identified as *loyalty, authority*, and *purity* and are associated with loyalty to one’s ingroups, such as family and country, deference to authority figures, such as elders, and an increased tendency to endorse concepts of purity and sanctity (Haidt, 2012). These moral values have collectively been named “*binding*” foundations because they are argued to bind communities and groups together (Graham et al., 2009; Haidt, 2012). The individualizing and binding foundations are said to have evolutionary origins and have important implications for a number of diverse political and cultural ideologies (Haidt and Joseph, 2004; Haidt, 2012). Crucially, however, the role of moral values for understanding intergroup relations is less clear and the relationship to strong social ideologies is still under-developed. This paper aims to show how individualizing and binding moral foundations have different relationships to intergroup variables of threat and attitudes and further aims to develop our understanding of the relationship to strong social ideological belief systems.

A number of studies have suggested that moral foundations may help explain strong ideological views concerning intergroup relations. Ideology is generally defined as “a more or less systematic ordering of ideas with associated doctrines, attitudes, beliefs, and symbols that together form a more or less coherent philosophy or Weltanschauung for a person, group, or sociopolitical movement” (American Psychological Association, 2022, p. 1). In the current research we examine two ideologies focused on *intergroup* relations and that represent particularly strong sets of beliefs and attitudes and will use the term ‘social ideologies’ to describe these constructs. These extreme or strong social ideologies include authoritarianism (RWA; Altemeyer, 1988, 1996) and social dominance orientation (SDO; Pratto et al., 1994), which are theoretically and empirically distinct and have a number of important implications for intergroup relations (Duckitt and Sibley, 2009, 2010). RWA comprises three main components based upon preference for heightened conformity to norms, submission to authority figures, and endorsement of aggression against outgroups that deviate from subscribed norms (Altemeyer, 1996). In contrast, SDO reflects preference for hierarchy and status-based societal relations, even if this has a negative impact on other groups or individuals with lower status. Individuals high in SDO also endorse beliefs that people act in a competitive way (Duckitt and Sibley, 2009, 2010). Overall, research has demonstrated that RWA and SDO represent distinct constructs (Duckitt and Sibley, 2009, 2010). Both of these ideologies also have important implications for intergroup relations because RWA and SDO predict increases in different types of prejudice and have been related to strongly negative outgroup attitudes across a large body of research (Pratto et al., 1994; Altemeyer, 1996; Duckitt and Sibley, 2007).

### Moral foundations and intergroup attitudes

The relationship between moral foundations, intergroup relations, and beliefs is not understood well, and the influence of moral foundations on intergroup relationships and strong social ideologies like RWA and SDO is only partially known. Early work has suggested that more endorsement of the individualizing foundations of harm and fairness relate to lower SDO scores (Federico et al., 2013; Kugler et al., 2014), which may reflect an anti-egalitarian stance of those high in SDO (Pratto et al., 1994; Federico et al., 2013). In contrast, more endorsement of the binding foundations of loyalty, authority, and purity are related to higher RWA scores, which may reflect the high emphasis on conformity to societal norms and traditions within authoritarian ideologies (see Koleva et al., 2012; Federico et al., 2013; Kugler et al., 2014; Hadarics and Kende, 2017). This research has deepened our understanding of fundamental moral values in the formation of these social ideological beliefs, but the relationship between moral foundations, intergroup attitudes, and threat perceptions is not understood as well (Kugler et al., 2014; Smith et al., 2014; Baldner and Pierro, 2019; Forsberg et al., 2019). Additionally, the mediational influence of RWA and SDO on the relationship between moral foundations and intergroup attitudes is largely untested though some researchers have examined the links between social ideologies such as RWA and SDO, and moral foundations (Federico et al., 2013; Kugler et al., 2014). One group of researchers have measured moral foundations, social ideologies, and attitudes together at the same time (Hadarics and Kende, 2018). This research has measured the influence of RWA and SDO on moral foundations as opposed to moral foundations predicting RWA and SDO and has noted how moral foundations have mediated the effects of RWA and SDO to some outgroup attitudes (i.e., derogated groups, dissident groups, and dangerous groups). Other researchers have investigated the association of RWA and SDO to moral foundations (Federico et al., 2013; Kugler et al., 2014) and Kugler showed that the binding foundations were associated with more outgroup hostility to groups (undocumented immigrants, Muslims) while individualizing foundations were associated with less hostility. One aspect of our research is to investigate moral foundations predicting attitudes, and RWA and SDO mediating this effect. Furthermore, research has been limited by measuring threat and inferring outgroup attitudes (Hadarics and Kende, 2017) or by measuring outgroup attitudes and inferring threat (Hadarics and Kende, 2018). In such cases, threat perceptions were not measured as a separate variable from intergroup attitudes meaning that it was not possible to measure the impact of threat perceptions upon more general intergroup attitudes in the context of the MFQ and social ideologies. Therefore, threat perceptions may have important unstudied implications for social and political psychology and for social interventions.

Given that moral foundations are proposed to be fundamental motivations, they may predict intergroup attitudes in addition to being related to RWA and SDO. Kugler et al. (2014) observed that the binding foundations of loyalty, authority, and purity positively correlated with hostility toward outgroups. While increased hostility was observed, the generality of this effect is still uncertain because the measure included a variety of outgroup comparisons that could be considered more salient for Americans (e.g., undocumented immigrants; Muslims compared to Christians). In other research, Smith et al. (2014) found that strong support for the binding foundations related to more support for strongly negative treatment toward outgroups (e.g., torture, willingness to share water resources), but *only* if participant’s moral identity was low; Smith et al. (2014), measured moral identity using a subscale concerned with participant’s moral internalization, reflecting how central morality is to an individual’s identity (Aquino and Reed, 2002). Further research has also found support for more militaristic solutions to conflict in those high in the binding foundations and more diplomatic/cooperation-orientated solutions for those high in the individualizing foundations (Kertzer et al., 2014), and that those high in the binding foundations were more likely to support ingroup-oriented charities such as medical research whereas those high in the individualizing foundations would support overseas aid for outgroups as well as ingroup focused charities (Nilsson et al., 2016). When considering attitudes toward the poor, both harm reduction and fairness were related to more positive attitudes while loyalty, authority, and purity were related to more negative attitudes toward people who are poor (Low and Wui, 2016). Finally, recent studies have shown that binding foundations are related to more negative attitudes toward immigrants and individualizing foundations predicted more positive attitudes toward immigrants, and that these relationships hold for a wider variety of disadvantaged groups (Baldner and Pierro, 2019; Forsberg et al., 2019; Stewart and Morris, 2021). Together these early research findings suggest overall differences in attitudes toward groups as a function of the individualizing and binding moral values. Our research attempts to test these relationships between individualizing and binding foundations and intergroup attitudes, and then test whether strong social ideologies such as RWA and SDO will mediate these relationships. Understanding these processes may further help to elucidate the value systems underlying these strong or more extreme attitudes.

In relation to this previous literature, a number of questions can be asked about the relationships between moral values, social ideologies, and attitudes. One group of researchers have focused on moral foundations mediating the relationship between RWA and attitudes and SDO and attitudes (Hadarics and Kende, 2018). Another research endeavor showed that RWA and SDO mediated the relationship between belief in a dangerous world and moral foundations, and between competitive jungle beliefs and moral foundations (Federico et al., 2013). However, none have investigated the moral foundations to attitudes relationship being mediated by RWA and SDO. Thus, there is further scope to consider whether a relationship between the binding foundations and intergroup attitudes would be mediated by RWA, and whether the relationship between individualizing foundations and intergroup attitudes is mediated by SDO. Previous work has shown that RWA is characterized by strong support for traditional group values, and that SDO is characterized by strong support for groups to be in hierarchies (Pratto et al., 1994; Altemeyer, 1996). For example, Federico et al. (2013) demonstrated that binding foundations were more consistently and strongly correlated to RWA than were individualizing foundations, while individualizing foundations were more strongly correlated with SDO than were binding foundations. Kugler et al. (2014) demonstrated similar relationships between binding foundations and RWA, and between individualizing foundations and SDO, though they also found SDO to be related to loyalty and authority. They further demonstrated a link between more endorsement of binding foundations and more outgroup hostility and others have shown that binding foundations are related to more negative attitudes and individualizing foundations are related to more positive attitudes toward immigrants, the poor, and a wide range of outgroups (Low and Wui, 2016; Baldner and Pierro, 2019; Forsberg et al., 2019; Stewart and Morris, 2021). In the current research, we aim to test whether endorsement of binding foundations are related to more negative attitudes toward immigrants and whether endorsement of individualizing foundations are related to more positive attitudes. Further, we sought to examine whether SDO would mediate the individualizing to intergroup attitude relationship and whether RWA would mediate the binding to intergroup attitude relationship.

Researchers have argued that moral foundations derive from intuitive responses to moral dilemmas and inform our attitudes (Haidt and Joseph, 2004; Haidt, 2012) and others have argued that SDO and RWA are social attitudes that may develop in late adolescence (Altemeyer, 1998; Van Hiel et al., 2004; Sibley and Duckitt, 2013). When considering the ordering in our research, we draw upon the previous research and upon MFT, which utilizes developmental, evolutionary, and cultural evidence to justify its placement as an antecedent to social attitudes (Haidt, 2012). In its development, MFT aimed to expand upon traditional models of morality such as those found in the work of Kohlberg (1969) and Gilligan (1982), which were primarily examining justice and care, respectively (Graham et al., 2009). Haidt and Joseph (2004) reviewed work that focused on community and divinity that had not been included in the morality literature in order to incorporate a broader swath of cross-cultural and anthropological research in their theory development (Shweder et al., 1997). Graham et al. (2009) and Haidt (2012) included these concepts into their binding foundations. Further research has demonstrated consistent patterns across the ideological and cultural spectrum in the endorsement of each moral foundation, which demonstrates a strong empirical basis for MFT in understanding ideological and policy positions (Haidt, 2007; Graham et al., 2009; Koleva et al., 2012). In regard to the ordering of variables, moral foundations are hypothesized to have evolved as fundamental moral values. For example, our intuitive reactions of concern when seeing a child in distress are a reflection of the harm foundation (Haidt, 2012). Similar mechanisms underlie the binding foundations. For example, our quickly activated disgust reactions are derived from disease avoidance and reflected in the purity foundation (Haidt, 2012). MFT argues that moral foundations are derived from these rapid, intuitive reactions and they inform our attitudes on social issues. According to the theory, if we wish to understand why people hold different political or social ideologies, we have to first understand the intuitive moral values and reactions that influence such attitudes (see Haidt, 2001 for the SIM Model; also Haidt, 2012). Thus, in our studies we placed moral foundations as predictors of the RWA and SDO ideologies, and utilized RWA and SDO as mediators. While we had good theoretical reasons to do this, we also empirically tested this ordering with structural equation models and found that our ordering had better fit than models using RWA and SDO as predictors (see Supplementary materials: ‘Alternative Order Analysis,’ Supplementary Tables S.3–S.5).

### The importance of threat processing in intergroup relations

When considering the implications of moral foundations for intergroup relations, there is a further reason to consider threat perceptions as an explanatory variable. While research has begun to consider the role of moral foundations in intergroup relations, the role of threat perceptions in explaining this relationship has not been investigated much despite the extremely strong relationship between threat perceptions and a number of relevant variables related to intergroup attitudes. For example, in early models of intergroup relations, objective and subjective threats to resources, to an ingroup’s existence, and to economic and material well-being, have all played a crucial role in creating intergroup conflict (Sherif, 1966; Levine and Campbell, 1972). In addition, symbolic threats to values and beliefs have been added to these models and have been linked to more negative intergroup attitudes and prejudice (Kinder and Sears, 1981; Esses et al., 1993; Stephan and Stephan, 1996). Recent research has also demonstrated a strong association between more symbolic and realistic threat and more prejudice (Stephan et al., 1999; Riek et al., 2006; Rios et al., 2018). While threat has been examined in the context of intergroup relations, it also has important implications for the study of moral foundations and more extreme social ideologies, such as RWA and SDO (Stenner, 2005; Cohrs and Ibler, 2009; Costello and Hodson, 2011).

Threat may play a larger role for the understanding of differences in social ideologies of RWA and SDO than previously thought. For example, preference toward binding foundations is positively related to belief in a dangerous world (Van Leeuwen and Park, 2009), which is a form of threat perception, and belief in a dangerous world has been found to also underlie social ideologies such as RWA (Altemeyer, 1988; Duckitt, 2006; Duckitt and Sibley, 2009, 2010). RWA has also been found in turn to predict viewing outgroups as threatening and to be strongly linked to higher levels of perceived threat to one’s group (Duckitt, 2006; Kauff et al., 2015; Duckitt and Sibley, 2017; Hadarics and Kende, 2017; Sinn and Hayes, 2017). Given the relationship of threat to both binding foundations and to intergroup attitudes, perceived threat may be a powerful mediator of the binding foundation to attitudes relationship. Furthermore, given the strong association between threat and RWA, perceived threat may play the largest role as an additional mediator of the binding foundation relationship. Importantly, threat may be connected to other ideologies beyond RWA. Numerous studies have connected higher SDO to higher threats to one’s ingroup resources and competition and that these are strong relationships (Morrison and Ybarra, 2008; Crowson, 2009; Uenal, 2016; Uenal et al., 2021). Uenal (2016) found a relationship between SDO and more threat including both realistic and symbolic threat and also demonstrated a relationship between higher SDO and more prejudice. Together these research findings support the idea that threat may be a powerful mediator of the relationship between individualizing foundations and intergroup attitudes and that it may play a larger mediational role than SDO.

In the current research, we were first interested in whether RWA would mediate the relationship between binding foundations and negative intergroup attitudes and whether SDO would mediate the relationship between individualizing foundations and less negative attitudes. We then sought to extend this research by testing whether threat explains variance over and above RWA and SDO mediators when examining the relationship between moral foundations and intergroup attitudes. Such a finding may elucidate the role of threat-based cognition in extreme attitudes. Since researchers have indicated that RWA and SDO are social attitudes that may be developed in life at a later stage and that moral foundations are thought to be intuitive responses that inform our attitudes, there is support for their placement as mediators in the current research (Hooghe and Wilkenfeld, 2008; Haidt, 2012; Sibley and Duckitt, 2013).

### Moral foundations, right-wing authoritarianism, social dominance orientation, and threat predictions

We predict that:

(1) More endorsement of individualizing foundations will relate to less SDO.
(2) The relationship between the individualizing foundations and less negative attitudes toward immigrants will be mediated by SDO.
(3) More endorsement of binding foundations will relate to higher RWA.
(4) The relationship between binding foundations and more negative attitudes toward immigrants will be mediated by RWA.

If statistical mediation occurs in the above models, we will add threat perceptions to each of the mediational models. We will investigate whether

(1) including a threat mediator will explain variance over and above the SDO mediator in the individualizing foundations to attitudes model
(2) including a threat mediator will explain variance over and above the RWA mediator in the binding foundations to attitudes model.

## Study 1

### Method

#### Design and procedure

The study employed a measurement of mediation regression design with the MFQ individualizing foundations and MFQ binding foundations as predictors, and attitudes toward immigrants and perceived threat as outcome variables. We followed previous research in using the individualizing foundations (average of harm and fairness) and binding foundations (average of loyalty, authority, and purity) as general indexes of moral orientations (Van Leeuwen and Park, 2009; Kidwell et al., 2013; Smith et al., 2014). SDO was the mediator for the individualizing to intergroup attitudes analysis, and right-wing authoritarianism (RWA) was the mediator for the binding to intergroup attitudes analysis based upon relationships observed in the literature. We used SDO and RWA as mediators instead of individualizing and binding as mediators because moral foundations are meant to be basic values, and we were interested in further examining the individualizing foundations to attitudes relationships and the binding foundations to attitudes relationships. To statistically control for order effects of the mediators, we created a number of counterbalanced orders of the mediators and of the outcome variables. We also included a perspective taking measure as an additional variable, though as this was not related to our primary topics of investigation, we include a brief rationale around perspective taking in Text Footnote 1 and further details are included in the methods and results sections. The study included four orders to which participants were randomly assigned (Order 1: MFQ-filler task-RWA-SDO-filler-Attitudes-Threat-Perspective Taking; Order 2: MFQ-filler task-SDO-RWA-filler-Attitudes-Threat-Perspective Taking; Order 3: MFQ-filler task-RWA-SDO-filler-Attitudes-Perspective Taking-Threat; Order 4: MFQ-filler task-SDO-RWA-filler-Attitudes-Perspective Taking-Threat). A numerical filler task and a reading filler task were included before and after the mediators in order to reduce participant suspicion. In addition, we used a measurement of mediation approach because past research has produced only small or weak effects of manipulating individualizing and binding focus and there are a lack of studies manipulating RWA and SDO ideologies previously (Kidwell et al., 2013; Day et al., 2014; Feinberg and Willer, 2015; Wolsko et al., 2016).

The MFQ predictor variables were administered first, followed by mediators, and then outcome variables as recommended for mediation analyses when the mediators are measured (Cohen et al., 2003). All participants received the MFQ first followed by the first filler task. After completion of the first filler task, participants in Orders 1 and 3 received RWA followed by SDO while those in Orders 2 and 4 received SDO followed by RWA; all participants then received a second, reading filler-task followed by the attitudes outcome measure. Attitudes were measured first because it was a short, five-item measure which may be more influenced by socially desirable responding than the longer, fifteen-item threat measure that asked about a larger variety of opinions. The threat and perspective taking measures were counterbalanced where participants in Orders 1 and 2 received threat followed by perspective taking and participants in Orders 3 and 4 received the perspective taking subscale followed by threat. After these measures were completed, all participants completed demographic measures including age, gender, and political orientation.

#### Participants

For Study 1, we used the average effect size found in personality and social psychology (*r* = 0.21) as a basis to generate our sample size (Richard et al., 2003; Gignac and Szodorai, 2016). This produced an estimated sample of approximately 170 participants for 0.8 power. A total of 172 participants completed the study using Amazon’s Mechanical Turk online platform in exchange for monetary compensation. Participant eligibility to take part in the study was based on a good performance rate for other studies and tasks on the MTurk platform. All participants who completed the study were located in the United States. We used a United States sample because it was the only sample to which we had access via an online format in MTurk (Buhrmester et al., 2011). As is standard in moral foundation research participants were removed for demonstrating acquiescence on two items that check for inattention (Graham et al., 2009). An example inattention item is answering that it is more than slightly relevant that someone is good at math “when you decide something is right or wrong.” This left a final sample of 157 participants with an age range from 21 to 61 (*M* = 32.92, *SD* = 9.12), and with 78.3% white and 39.5% female.

#### Materials

##### Moral foundations questionnaire

To measure participants’ investment in moral issues, we used the moral foundations questionnaire (MFQ) that consisted of 32 items with two items used to ensure participants are paying attention while completing the scale (Graham et al., 2008). The remaining 30 items assessed investment in the five moral foundations of harm, fairness, loyalty, authority, and purity. Each moral foundation (e.g., harm) consisted of six items that were averaged to form an overall score for each foundation with items within the MFQ relevance and judgment subscales presented in a random order to participants. The computer program coded the relevance subscales of the MFQ as 1 = *Not at all relevant*, and 6 = *Extremely relevant*, and the judgment subscales were coded 1 = *Strongly disagree*, and 6 = *Strongly agree*. The binding foundation score (*M* = 3.28, *SD* = 0.92, α = 0.92) was created by averaging loyalty, authority, and purity scores and the individualizing foundation score (*M* = 4.60, *SD* = 0.67, α = 0.79) was calculated by averaging harm and fairness scores (Van Leeuwen and Park, 2009).

##### Filler task 1 (numerical filler task)

To act as a buffer between the MFQ measure and the mediators (i.e., RWA and SDO), we used a filler task termed a “short task of cognitive processing.” Participants were told that they would select the number indicated from a list of numbers. They were asked to be as fast and accurate as possible and that in each trial they would be asked to click on a target number among 9 other distractor numbers which varied throughout the task. They then completed 40 trials in a list format with the target number changing on each trial and the order of the distractor numbers (i.e., 1 through 10 or 10 through 1) changing by trial. The task was designed to be simple and engaging so as to act as a delay between sections of the study.

##### Right-wing authoritarianism short-form scale

To measure investment in authoritarian ideology, we employed the 15-item version of the RWA scale from Zakrisson (2005). Participants responded to statements such as “*If the society so wants, it is the duty of every true citizen to help eliminate the evil that poisons our country from within.*” Responses were completed on a seven-point Likert scale labeled from (1) *Very Negative* to (7) *Very Positive*. Items within the RWA scale were presented to participants in a random order. After reverse scoring seven items, the scale was averaged with higher scores reflecting higher authoritarian ideology (*M* = 2.78, *SD* = 1.17, α = 0.92).

##### Group-based social dominance orientation

To measure participant level of SDO toward groups, we used the group-based, 16-item version of Pratto et al.’s (1994) SDO scale. It includes such items as: “*To get ahead in life, it is sometimes necessary to step on other groups.*” All items were completed on a seven-point Likert scale ranging from (1) *Very Negative* to (7) *Very Positive*. Items within the SDO scale were presented to participants in a random order. After reverse scoring eight items, the scale was averaged with higher scores reflecting higher social dominance ideology (*M* = 2.13, *SD* = 1.18, α = 0.96).

##### Filler task 2 (the growing stone task)

We used the growing stone task that has been included in other research as a delay task (Greenberg et al., 1994); it acted as a second filler task between the mediators and the main outcome variable of the study. In this filler task, participants read a literary passage and then rated the prose in terms of a number of features; in our version, we removed any reference of race. Participants were first asked to rate the passage in terms of “*How do you feel about the overall descriptive qualities of the story?*” providing a rating on a nine-point scale ranging from (1) *not at all descriptive* to (9) *very descriptive*. Participants also rated how engaging and imaginative they found the story using a nine-point scale.

##### Intergroup attitudes

To assess participant’s attitudes, we used five items adapted from Saguy et al. (2009). Participants were asked to rate their feelings toward immigrants on five evaluative dimensions (i.e., warmth, negativity, friendliness, suspicion, and admiration) on nine-point scales with a neutral midpoint (i.e., 1 = *Extremely Warm* to *9* = *Extremely Cold*). Two items were reverse scored and the five items were averaged to form a measure of attitudes with higher scores reflecting more negative attitudes toward immigrants (*M* = 3.95, *SD* = 1.66, α = 0.96).

##### Threat perceptions

Perceived threat from immigrants was measured using a scale by Stephan et al. (1999). The measure comprises two subscales regarding realistic threat, “*Immigration has increased the tax burden on Americans*,” and symbolic threat, “*The values and beliefs of immigrants regarding social relations are NOT compatible with the beliefs and values of most Americans.*” Items were completed on a seven-point scale (1) *Disagree Strongly* to (7) *Agree Strongly* with neutral midpoint. Eight items were reverse scored and the 15 items were then averaged. Given the high correlation between symbolic and realistic threat (*r* = 0.814, *p* < 0.001) and the use of a collapsed scale to assess threat in prior research, we collapsed symbolic and realistic subscales (Tausch et al., 2007; Verkuyten, 2009; Green et al., 2010; Tip et al., 2012; Schmid and Muldoon, 2015; Stewart et al., 2019). We then computed a measure of overall perceived threat toward immigrants, all items were averaged to form a measure of threat following previous research (*M* = 3.18, *SD* = 1.39, α = 0.96).

##### Perspective taking subscale

We used the cognitive perspective taking subscale of the interpersonal reactivity index (IRI: Davis, 1983) which consists of seven items assessing cognitive perspective taking (PT), including items such as “*I try to look at everybody’s side of a disagreement before I make a decision.*” Responses were scored on a five-point scale from (1) *Does not describe me well* to (5) *Describes me very well*. Two items were reverse scored and the scale was averaged to form an overall index of perspective taking (*M* = 4.06, *SD* = 0.72, α = 0.87). We include a brief rationale for our inclusion of perspective taking items in footnote 1. Results from perspective taking analyses are included in the linear regression tables in the results section, across studies.

##### Mathematics items

We included math items as a test of participant attention with participants who provided incorrect responses for all items being excluded. Only one participant answered all items incorrectly however this participant had already been excluded based on the attention check item within the MFQ.

##### Demographics

After completion of all of the main items in the study, participants then completed demographic items including age, gender, ethnicity, number of years speaking English and number of years living in the United States, political ideology (Graham et al., 2009; Haidt et al., 2009), and level of education. All participants had lived in the United States for nine or more years.

##### Self-construal scale

The self-construal scale by Singelis (1994) was included as a pretest for another study and was included after all other study variables were administered in order to have no influence on the main study. Thus, it was not included in the analyses.

### Results

#### Moral foundations and social ideology

We first examined the relationships between the individualizing (average of harm and fairness) and binding (average of loyalty, authority, and purity) foundation indexes and the intergroup variables of interest. We conducted three separate linear regressions for the individualizing index predicting each of the main outcome measures (attitudes, threat, and perspective taking) and three separate linear regressions for the binding index predicting each of three outcome variables. We used linear regressions with just one index, instead of multiple regressions, because we were interested in each of the indexes relationships to the outcome variables and were not interested in determining how one related to the outcomes in the presence of the other index. In these analyses higher scores on the attitudes variable represented more negative intergroup attitudes. The relationships between moral foundations and these variables can be found in Table 1.

**TABLE 1:** Linear regression analyses of moral foundation indexes to outcomes for Study 1.

	Separate linear regressions				Bootstrapping		
	β	*P-value*	*R* ^2^	*t*	*b*	*95% CI for b*	*P-value*
**Predictor: Individualizing foundations**							
**Outcome:**							
Perspective taking	0.26	0.001	0.07	3.37	0.28	[0.11, 0.47]	0.002
Attitudes	−0.23	0.003	0.05	−2.99	−0.58	[−0.97, −0.15]	0.006
Threat	−0.24	0.002	0.06	−3.10	−0.50	[−0.81, −0.14]	0.004
**Predictor: Binding foundations**							
**Outcome:**							
Perspective taking	−0.02	0.83	<0.01	−0.22	−0.01	[−0.13, 0.11]	0.81
Attitudes	0.37	<0.001	0.14	4.94	0.67	[0.39, 0.95]	<0.001
Threat	0.50	<0.001	0.25	7.17	0.75	[0.53, 0.96]	<0.001

Higher scores for attitudes represent more negative intergroup attitudes, the individualizing foundations predictor represents the average of harm and fairness moral foundations, the binding foundations predictor represents the average of loyalty, authority and purity foundations.

**FIGURE 1:**
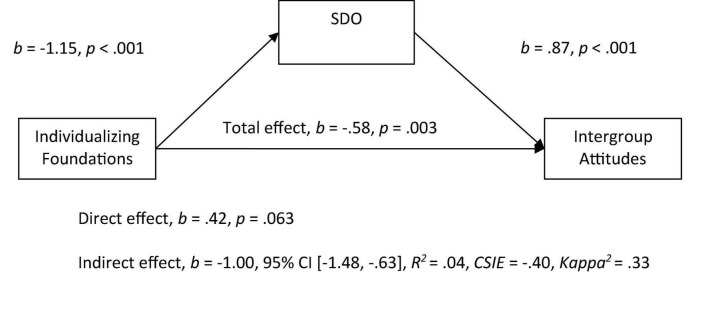
Mediation model of the relationship between individualizing foundations to intergroup attitudes by social dominance orientation. All betas represent unstandardized values from the Bootstrap analysis with 5,000 samples. CSIE represents the completely standardized indirect effect.

Mediational analyses were then conducted, using PROCESS for SPSS as suggested by Hayes (2013). Both the individualizing foundations (*M* = 4.60, *SD* = 0.67, α = 0.79) and the binding foundations had good reliability (*M* = 3.28, *SD* = 0.92, α = 0.92). In Model 1, we entered the individualizing foundation score (average of harm and fairness) as a predictor variable and attitudes toward immigrants as the outcome variable, and entered SDO as the mediator. As expected, we observed a significant indirect effect of individualizing foundations on intergroup attitudes toward immigrants through the SDO variable in which more endorsement of individualizing foundations related to lower SDO scores and higher SDO scores being related to more negative attitudes (see Figure 1). When SDO was included in the model as the mediator variable, the relationship between individualizing values and attitudes became non-significant.

**FIGURE 2:**
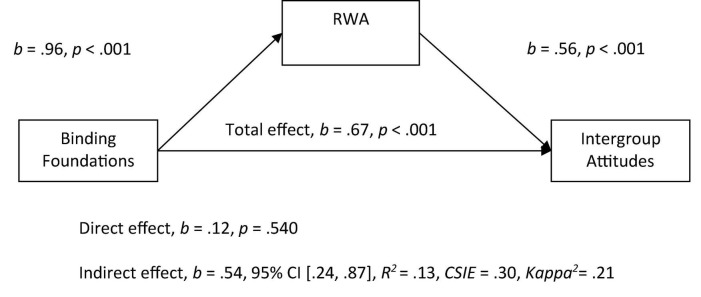
Mediation model of the relationship between binding foundations to intergroup attitudes by right-wing authoritarianism (RWA). All betas represent unstandardized values from the Bootstrap analysis with 5,000 samples. CSIE represents the completely standardized indirect effect.

In the separate Model 2, we explored the relationship between binding foundations and the prediction of more negative attitudes toward immigrants. The binding foundations score (average of loyalty, authority and purity) was entered as the predictor variable, and attitudes as the outcome variable, RWA was entered as the mediator. As expected, we observed a significant indirect effect of binding foundations on intergroup attitudes through RWA, in which higher endorsement of the binding foundations positively related to RWA scores, and with higher RWA scores relating to more negative attitudes (see Figure 2). Once RWA was included as a mediator in this model, the relationship between the binding foundations and attitudes became non-significant.

Some researchers have suggested that the social-political attitudes of RWA and SDO may be exogenous and predict moral foundations (Federico et al., 2013) instead of moral foundations predicting RWA and SDO (Sibley and Duckitt, 2013). We conducted structural equation modeling analyses to support our model ordering across studies and these analyses are included in our Supplementary materials (see Supplementary: ‘Alternative Order Analysis,’ Supplementary Tables S.3–S.5).

#### Exploratory analyses considering the role of threat

**FIGURE 3:**
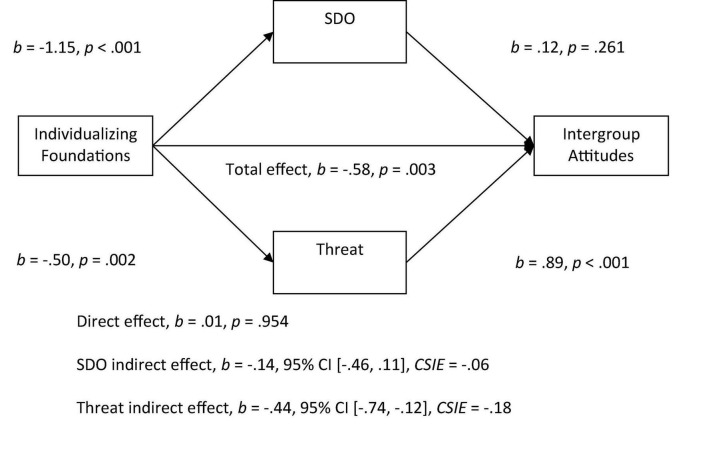
Mediation model of the relationship between individualizing foundations to intergroup attitudes by social dominance orientation and threat. All betas represent unstandardized values from the Bootstrap analysis with 5,000 samples. CSIE represents the completely standardized indirect effect.

Given that previous research has indicated that perceptions of symbolic and realistic threat are an important antecedent to prejudice, we tested whether perceived threat would be a significant mediator in addition to SDO for the individualizing to intergroup attitudes relationship. We did a similar test for the binding to attitudes relationship with RWA and threat as mediators (Riek et al., 2006; Duckitt and Sibley, 2009). With both SDO and threat in the model, we found a significant indirect effect of the individualizing foundations on attitudes through threat. More endorsement of individualizing foundations was related to less perceived threat and more threat was related to negative attitudes (see Figure 3). However, there was a non-significant indirect effect of SDO with both threat and SDO in the model.

**FIGURE 4:**
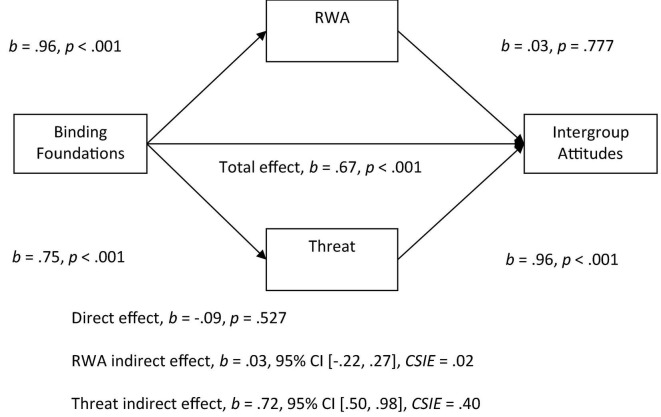
Mediation model of the relationship between binding foundations to intergroup attitudes by right-wing authoritarianism (RWA) and threat. All betas represent unstandardized values from the Bootstrap analysis with 5,000 samples. CSIE represents the completely standardized indirect effect.

With both RWA and threat in the model, we observed a significant indirect effect of binding foundations on attitudes through threat. More endorsement of the binding foundation was related to more perceived threat, which was related to more negative attitudes (see Figure 4). There was a non-significant indirect effect of RWA with both perceived threat and RWA in the model.

### Discussion

The mediation models demonstrated that strong social ideologies such as SDO and RWA are related to individualizing and binding foundations, respectively, as predicted by past research. The current study was the first to extend this research by showing that these extreme, social ideologies also provide one explanation for the connection between moral foundations and intergroup attitudes. The link between more endorsement of individualizing foundations and lower social dominance was associated with less negative attitudes toward immigrants, and the link between more endorsement of binding foundations and higher authoritarianism was associated with more negative attitudes toward immigrants. Threat processes also appear to play an important role in understanding strong social ideologies because including perceived threat in the mediations reduced the effects of RWA and SDO in these analyses. These findings extend previous work showing that moral foundations and strong social ideologies can be used to understand both attitudes toward outgroups and threat perceptions toward outgroups. Crucially, the role of threat in such phenomena should play a more focal role in future studies of moral and socio-political ideology and intergroup attitudes.

## Study 2

In Study 1, we demonstrated that the negative relationship between individualizing foundations and attitudes was mediated by SDO and the relationship between binding foundations and attitudes mediated by RWA. We also conducted an exploratory analysis including threat perceptions as a second mediator in each model; we observed that threat perceptions explained variance over and above SDO in the individualizing to attitudes model, and above RWA in the binding to attitudes model. While these findings were of note, they were exploratory and we wished to replicate and extend these findings in a confirmatory Study 2. Additionally, one limitation of Study 1 was the measure of threat perceptions and the outcome measure of attitudes were of the same group (attitudes toward immigrants). In Study 2, we wanted to reduce the impact of this common target variance in the threat and the attitude measures. In order to address this potential issue, Study 2 used a different group in the threat measure (immigrants) than in the outcome measures of both attitudes and negative attitudes (Mexican Americans, who may be thought of as immigrants, but are not predominantly immigrants; Noe-Bustamante et al., 2019; U.S. Census Bureau, 2019). A replication of our findings with different groups in the threat (mediator) and the attitudes measures (outcome variables) would serve to strengthen the robustness of our findings and the importance of more generalized notions of intergroup threat impacting upon attitudes. In addition, Study 2 counterbalanced the order of threat and attitudes, however, the attitudes measure always preceded the newly added negative attitudes measure to avoid contaminating the less negative measure. We also aimed to replicate our more basic models between the moral foundations, SDO, RWA, and intergroup attitudes as well as considering the impact of threat on these processes. Finally, Study 2 used a larger sample and a different online recruitment platform than was used in Study 1.

### Method

#### Design and procedure

In Study 2, we used similar methodology to Study 1 in which individualizing and binding foundations were predictor variables, RWA and SDO, and threat were the mediator variables, and intergroup attitudes and negative attitudes toward Mexican Americans were outcome variables. Mexican Americans represent a growing and important population in the United States and 71% of Mexican-Americans were born in the United States (Noe-Bustamante et al., 2019; U.S. Census Bureau, 2019). Participants first completed the moral foundations questionnaire followed by the number filler task from Study 1. Participants were then randomly assigned to one of 12 orders (see Supplementary Table S.6) in which the order of RWA and SDO were first counterbalanced, and then participants completed the prose filler task from Study 1. They then completed the threat, attitudes, and perspective taking measures in a counterbalanced order. This counterbalancing ensured that the key study variables of threat and attitudes occurred an equal amount of time before the other one; see Supplementary Table S.6. Finally, participants completed the demographics measures from Study 1 and were then debriefed and monetarily compensated for completion.

#### Participants

Given that we had a few smaller effect sizes (*R*^2^ = 0.01 and 0.02) in Study 1, we decided to obtain a larger sample in Study 2 to improve the ability to reliably observe these effect sizes. In Study 2, we aimed to get a large enough sample for 0.8 power for an effect size in the range of *R*^2^ = 0.02. A total of 431 participants were recruited from the United States using the Prolific.co online recruitment platform. Participant eligibility to take part in the study was based on a good performance rate for other studies and tasks on the Prolific platform. We removed participants who were of Mexican American ethnicity (*N* = 6) and participants who failed the attention check items on the MFQ (Graham et al., 2009). The final sample consisted of 388 participants with an age range of 18–76 (*M* = 32.17, *SD* = 12.07) of which 50.0% were female and 77.1% were white.

#### Materials

##### Moral foundations questionnaire

All participants first completed the same MFQ used in Study 1 (Graham et al., 2008) (individualizing *M* = 4.57, *SD* = 0.60, α = 0.74; binding *M* = 3.29, *SD* = 0.82, α = 0.88).

##### Number selection filler task

Study 2 used a version of the number selection filler task from Study 1 to act as a delay after the MFQ measure. Due to time restraints in the current study, the task was shortened to 30 rather than 40 trials. Again, each trial consisted of selecting one target number amongst nine other distractor numbers.

##### Right-wing authoritarianism and social dominance orientation

We used the same 15-item RWA scale and the same 16-item SDO scale as was used in Study 1 (RWA: *M* = 2.87, *SD* = 1.11, α = 0.91; SDO: *M* = 2.21, *SD* = 1.06, α = 0.94).

##### Growing stone delay task (filler 2)

As was done in Study 1, participants completed a filler task after completing the RWA and SDO measures, which has been used in previous research (Greenberg et al., 1994).

##### Intergroup attitudes

We also used the same measure of attitudes as was used in Study 1 (Saguy et al., 2009), but adapted the focal group to be Mexican Americans rather than immigrants. Higher scores indicated higher levels of negative attitudes toward Mexican Americans (*M* = 3.48, *SD* = 1.40, α = 0.93).

##### Negative attitudes

As an additional measure of negative attitudes toward Mexican Americans, we adapted a negative-focused attitudes measure from research by Stephan et al. (2002). The scale measured only negative sentiment and included five items assessing levels of disapproval, resentment, dislike, disdain, and hatred; the scale items used a ten-point scale with endpoints changing depending on the construct being measured and were scored from 0 “*no _____ at all” (e.g., no dislike at all) to* 9 *“Extreme ______” (e.g., Extreme dislike)*. These items were coded from 1 to 10 and had high reliability (*M* = 1.94, *SD* = 1.62, α = 0.96).

##### Threat perceptions and perspective taking

We used the same measure as was used in Study 1 to measure perceived threat from immigrants, as opposed to Mexican Americans; higher scores indicated higher perceived threat (*M* = 3.09, *SD* = 1.29, α = 0.94). We also used the same measure of perspective taking as was used in Study 1 (Davis, 1983; *M* = 3.83, *SD* = 0.77, α = 0.84)^1^. After completing the main outcome measures participants completed demographic questions.

### Results

#### Moral foundations and attitudes

In Study 2, we again examined the linear relationships between moral foundations and intergroup variables. We conducted four separate linear regressions for the individualizing index predicting each of the main outcome measures (attitudes, negativity, threat, and perspective taking) and four separate linear regressions for the binding index predicting each of the outcome variables. We also included a new negative-focused attitudes measure in this study and observed that individualizing foundations negatively and significantly predicted negative-focused attitudes, while binding foundations positively and significantly predicted negative-focused attitudes (see Table 2 for linear regressions).

**TABLE 2:** Linear regression analyses of moral foundation indexes to outcomes for Study 2.

	Separate linear regressions				Bootstrapping		
	β	*P-value*	*R* ^2^	*t*	*b*	*95% CI for b*	*P-value*
**Predictor: Individualizing foundations**							
**Outcome:**							
Perspective taking	0.18	<0.001	0.03	3.56	0.23	[0.08, 0.38]	0.003
Attitudes	−0.29	<0.001	0.09	−6.00	−0.68	[−0.93, −0.41]	<0.001
Negative attitudes	−0.17	0.001	0.03	−3.30	−0.45	[−0.79, −0.13]	0.007
Threat	−0.26	<0.001	0.07	−5.31	−0.56	[−0.77, −0.34]	<0.001
**Predictor: Binding foundations**							
**Outcome:**							
Perspective taking	−0.13	0.008	0.02	−2.67	−0.13	[−0.22, −0.04]	0.006
Attitudes	0.22	<0.001	0.05	4.35	0.37	[0.19, 0.56]	<0.001
Negative Attitudes	0.33	<0.001	0.11	6.77	0.65	[0.47, 0.84]	<0.001
Threat	0.52	<0.001	0.27	11.83	0.82	[0.68, 0.97]	<0.001

Higher scores for attitudes represent more negative intergroup attitudes, the individualizing foundations predictor represents the average of the harm and fairness foundations, the binding foundations predictor represents the average of loyalty, authority and purity foundations.

**FIGURE 5:**
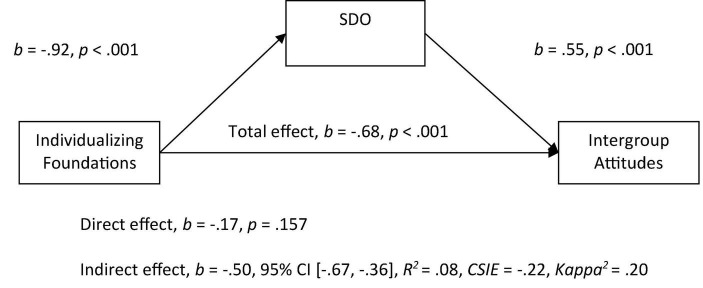
Mediation model of the relationship between individualizing foundations to intergroup attitudes by social dominance orientation. All betas represent unstandardized values from the Bootstrap analysis with 5,000 samples. CSIE represents the completely standardized indirect effect.

Following the methodological approach outlined in Study 1, we conducted mediations using the PROCESS macro (Hayes, 2013). In Model 1, we entered the individualizing foundations as the predictor variable and intergroup attitudes as the outcome variable while entering SDO as the mediator. Replicating Study 1 results, we observed a significant indirect effect of SDO (see Figure 5). More endorsement of individualizing foundations was related to lower levels of SDO, and higher levels of SDO was related to higher levels of negative attitudes toward Mexican Americans. When SDO was included as a mediator in the model the relationship between the individualizing foundations and attitudes became non-significant.

**FIGURE 6:**
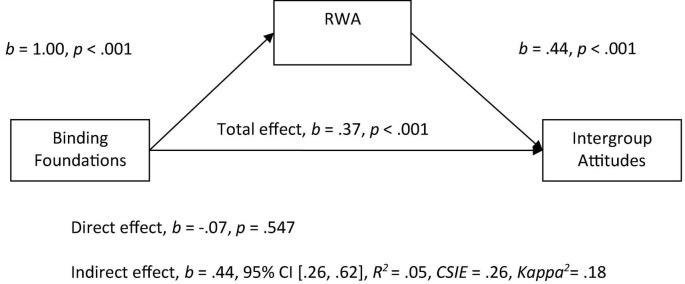
Mediation model of the relationship between binding foundations to intergroup attitudes by right-wing authoritarianism (RWA). All betas represent unstandardized values from the Bootstrap analysis with 5,000 samples. CSIE represents the completely standardized indirect effect.

In model 2, the binding foundations were entered as a predictor variable and intergroup attitudes acted as the outcome variable while entering RWA as the mediator. Replicating Study 1 results, we observed a significant indirect effect of RWA with more endorsement of binding foundations related to higher levels of RWA, which related to more negative attitudes toward Mexican Americans (see Figure 6). Including RWA as the mediator in the model caused the relationship between binding foundations and attitudes to become non-significant.

Following the methods outlined in Study 1, we also produced structural equation models (SEM) to justify the use of the moral foundations as predictor variables (see Supplementary materials: ‘Alternative Order Analysis,’ Supplementary Table S.4).

#### The role of threat perceptions in moral foundations, social ideologies, and intergroup attitudes

**FIGURE 7:**
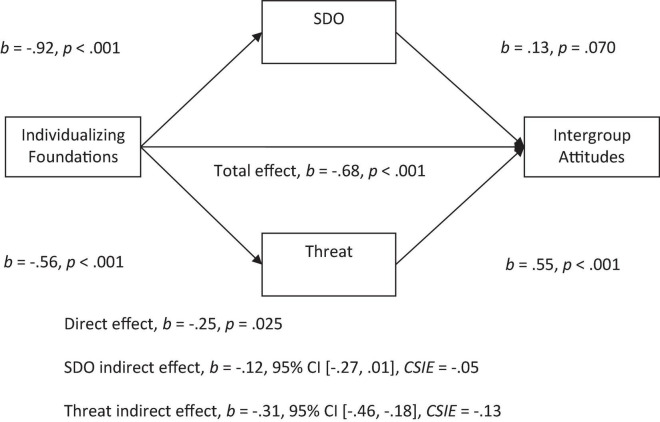
Mediation model of the relationship between individualizing foundations to intergroup attitudes by social dominance orientation and threat. All betas represent unstandardized values from the Bootstrap analysis with 5,000 samples. CSIE represents the completely standardized indirect effect.

We again employed a model in which threat was a second mediator alongside the mediator of SDO in the individualizing to intergroup attitudes (model 1), or threat was alongside RWA as a second mediator in the binding to intergroup attitudes (model 2). In the current study, intergroup attitudes were measured toward Mexican Americans (Noe-Bustamante et al., 2019; U.S. Census Bureau, 2019) and perceived threat was measured toward immigrants (non-specified group). This differed from Study 1 that measured attitudes toward immigrants and also measured perceived threat from immigrants. Including threat alongside the SDO mediator yielded a significant indirect effect of threat in which more endorsement of individualizing foundations was related to lower levels of perceived threat from immigrants, and more threat being related to more negative attitudes toward Mexican Americans (see Figure 7). This result replicated the Study 1 effect in which the indirect effect of the SDO mediator became non-significant when threat was entered in the model alongside SDO.

**FIGURE 8:**
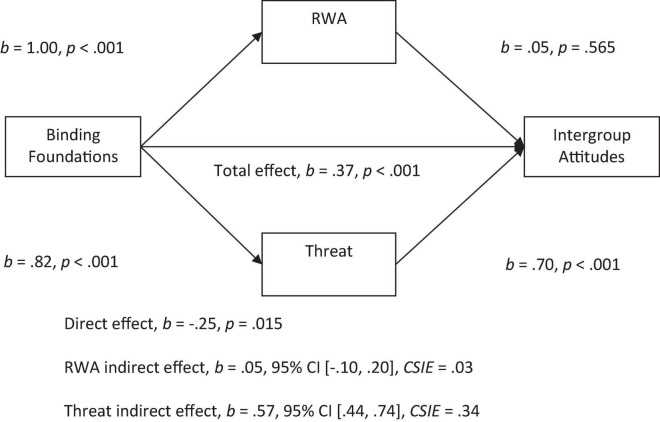
Mediation model of the relationship between binding foundations to intergroup attitudes by right-wing authoritarianism (RWA) and threat. All betas represent unstandardized values from the Bootstrap analysis with 5,000 samples. CSIE represents the completely standardized indirect effect.

In a separate model (see Figure 8), we included RWA and threat in the model as mediators between binding and intergroup attitudes toward Mexican Americans; we observed a significant indirect effect of the threat mediator in which more endorsement of binding foundations was related to higher levels of threat, which was related to more negative intergroup attitudes (see Figure 8). Again, this replicated Study 1 in which the indirect effect for the RWA mediator became non-significant when threat was included in the model alongside RWA.

#### Models using negative-focused attitudes as the outcome variable

In Study 2, we used an additional outcome variable of negative-focused attitudes toward Mexican Americans. This outcome measure assessed only negative sentiment (i.e., no reverse-scored items, with positive poles and a higher score representing more negative sentiment) in order to test the relationship toward a measure with only negative valence. We first ran the same set of mediational analyses performed for the intergroup attitudes models but this time using negative-focused attitudes as the outcome variable. In these analyses we replicated the effects observed in models 1 and 2, which had used attitudes as the outcome variable. We then conducted multiple mediation models again using negative-focused attitudes as the outcome and additionally including threat as a mediator entered at the same level as SDO and RWA, respectively. In these models, we also replicated our earlier attitudes models (models 3 and 4) from Study 1 and 2 (see the Supplementary Appendix for negative attitudes Mediation Models: Supplementary Figures A1–A4).

### Discussion

Study 2 replicated the general patterns detected in Study 1 and extended these findings by demonstrating the relationship between moral foundations and attitudes toward a different societal group (Mexican Americans). The only difference between Study 1 and 2 was the significant, but small (*R*^2^ = 0.02) negative relationship between binding and perspective taking, which was not significant in Study 1. Study 2 also included a second measure that tapped only negative-focused attitudes and this measure replicated the effects observed for the measure of general attitudes that included positive and negative items. Interestingly, the binding relationship was much stronger to negative-focused attitudes (*R*^2^ = 0.11) than to general attitudes (*R*^2^ = 0.05), which appears to match research by Kugler et al. (2014) using strong salient outgroup comparisons; this demonstrates the usefulness of including a measure using only negative items, some of which were very negative (i.e., hatred). Moreover, in Study 2 we replicated the same pattern of findings for the mediations that we had observed in Study 1. We found that the relationship between individualizing foundations and attitudes was mediated by SDO, and in a separate model, the relationship between binding foundations and attitudes was mediated by RWA; this was observed for both general intergroup attitudes and for negative attitudes, and toward a different social group, Mexican Americans.

In Study 2, we confirmed the exploratory analyses of Study 1 with perceived threat as a second mediator alongside RWA, or alongside SDO in these two, separate models. We found a significant indirect effect of the relevant MFQ variables through threat in explaining intergroup attitudes and negative-focused attitudes toward Mexican Americans in all cases. With threat entered alongside RWA, the indirect effect of binding foundations on attitudes, and binding foundations on negative-focused attitudes through the RWA mediator was non-significant in both cases, while the relationship through the threat mediator was significant in both cases. With threat entered alongside SDO, the indirect effect of the individualizing foundations on attitudes and individualizing on negative-focused attitudes through the SDO mediator was non-significant in both cases, while the relationship through the threat mediator was significant in both cases. These results suggest that threat processes may play a critical role in understanding strong social ideologies as well as moral foundations and different forms of biases.

In Study 2, we also ensured that the outcome measure of attitudes and the measure of threat perceptions did not measure attitudes toward the same groups (attitudes toward Mexican Americans and threat from Immigrants, respectively). This procedure reduced the potential issue of common-target variance in explaining the effect in our earlier study (Study 1). Importantly, this effect was observed even when attitudes toward Mexican Americans was measured before threat from immigrants (*N* = 190); thus, the effect remains even when thinking of Mexican Americans as opposed to being primed to think of immigrant Mexican Americans. Overall, Study 2 replicated the general patterns of Study 1 using attitudes toward a different group (Mexican Americans) and additionally using a measure focused on negative attitudes which replicated the patterns detected when considering intergroup attitudes (see Appendix for negative attitudes mediation models). These results suggest important relationships between moral foundations, social ideologies, and different forms of bias which may be important in understanding the causes of bias toward immigrant groups and also in explaining attitudes toward racial groups more generally. Crucially in a second study we replicated our threat findings from Study 1 for both general intergroup attitudes and negative attitudes, which suggests the importance of threat-based cognition in the understanding of these relationships.

## General discussion

The current research provides consistent evidence that moral foundations play an important role in understanding perceptions of societal groups and that moral foundations also have a strong association with social-political ideologies of RWA and SDO. Our findings further suggest the importance of threat perceptions in understanding these relationships. In Study 1, we found that endorsement of the individualizing foundations (i.e., average of harm and fairness) was associated with significantly less negative attitudes and less perceived threat and that endorsement of binding foundations (i.e., average of loyalty, authority, and purity) was significantly related to more perceived threat and more negative attitudes toward immigrants. The current research was also one of the first pieces of research that examined the relationships of individualizing and binding foundations to outgroup attitudes *and* the role that perceived symbolic and realistic threat played in mediations with RWA and SDO, which built upon one paper that showed that moral foundations mediated the relationship between RWA and outgroup attitudes and SDO and outgroup attitudes (Hadarics and Kende, 2018). Some other researchers have shown associations between RWA and moral foundations, and SDO and moral foundations (Federico et al., 2013) and others have shown that binding foundations were associated with more outgroup hostility, and individualizing foundations were associated with less hostility (Kugler et al., 2014). Moreover, this was the first time that the individualizing foundations to intergroup attitudes association had been shown to be mediated by SDO and the first time that the binding foundations to intergroup attitudes association had been shown to be mediated by RWA; both of these mediations became non-significant once perceived threat was included in the bootstrapped, multiple-mediation models, which emphasizes the importance of threat perceptions. This was also the first time that threat perceptions had been included in the mediations for the moral foundations to attitudes relationships. In Study 2, we replicated the associations with attitudes and the mediations, including the mediations by threat. Study 2 also extended the findings to focus on an ethnic minority group (Mexican

Americans) while also including a measure of attitudes with only negatively focused items; in particular, these patterns were replicated even when attitudes toward Mexican Americans were measured prior to threat from immigrants; therefore, it was unlikely that participants were primed to think of Mexican Americans as just immigrants with this order of variables, and thus, unlikely to conflate the relationship. It, however, should be noted that we cannot be sure how participants interpreted the term Mexican Americans in our study because we did not assess their interpretation of the group. However, it appears that the same patterns of intergroup attitudes may relate to both immigrants and Mexican Americans.

The observed pattern of results shed light on how underlying moral values may help account for intergroup beliefs and attitudes and further suggests that moral foundations could be used to contribute to ways of understanding strong social ideologies such as RWA and SDO. The current research also highlights potential avenues for improving negative intergroup attitudes more generally by addressing groupings of moral values. Research on moral framing of issues has begun to hint that framing policies with moral foundations may persuade people who invest in the relevant foundations (Feinberg and Willer, 2013; Kidwell et al., 2013). Unfortunately, this research is in its early stages and has often produced very small or weak effects of the framing changing attitudes and can also cause entrenchment of existing beliefs instead of attitude change (Kidwell et al., 2013; Day et al., 2014; Feinberg and Willer, 2015; Wolsko et al., 2016). It has been difficult to effectively manipulate or frame these variables. Moreover, this research has often focused on framing individual moral foundations (e.g., harm, purity, etc.) rather than the individualizing and binding foundations, with the exception of a few studies that have begun investigating broader frames and factors (Kidwell et al., 2013; Wolsko, 2017; Hurst and Stern, 2020).

One promising line of research conducted by Mooijman et al. (2018) has used the approach of first demonstrating a consistent relationship between the binding moral foundations and perceptions of self-control (as a moral issue) using correlational methods. After the correlational relationship had been established, they then utilized experimental manipulations to establish causality based upon work within cultural mindset theory (see Oyserman and Lee, 2008). In three experiments, Mooijman et al. (2018) made either the binding (or individualizing) foundations more salient by adapting experimental mindset manipulations involving focusing on the moral characteristics of a fictional character, or in a different manipulation by completing part of an essay-based statement in support of a target set of moral values (adapted from Oyserman and Lee, 2008). These manipulations in turn led to higher ratings of moralization of self-control as an outcome variable for participants in the binding salience condition relative to participants in the individualizing salience condition. While much of the framing research is in its early stages, approaches such as those employed by Mooijman et al. (2018) may lead to the capacity for future research to demonstrate causal relationships between moral foundations and perceptions of different groups using experiments in which binding foundations or individualizing foundations are made salient. Our current research is limited because of the use of mediation models and establishing correlations between moral foundations, social ideologies, and intergroup attitudes. Future work using experimental approaches such as those used by Mooijman et al. (2018) may represent promising methods for establishing causality regarding moral foundations, ideologies, and intergroup attitudes.

Overall, we observed a consistent pattern across both studies regarding moral foundations and perceptions of intergroup threat. Endorsement of individualizing moral foundations was consistently associated with *less* perceived threat while endorsement of binding moral foundations was consistently related to *more* perceived threat. The link with binding foundations is in accordance with other research showing that more investment in binding is related to more endorsement of belief in a dangerous world or RWA, both of which are constructs that are related to threat perceptions (Duckitt and Sibley, 2009; Van Leeuwen and Park, 2009; Federico et al., 2013; Hadarics and Kende, 2017). Our findings are also consistent with recent research linking more endorsement of binding foundations with more outgroup threat for outgroups perceived as very non-normative (Sinn and Hayes, 2017). Thus, it appears that perceptions of symbolic and realistic threat may be key factors in the relationship between moral values and intergroup bias^2^. These threat perceptions may relate to a difference in risk acceptance, which may provide an avenue for discussions that focus on how to balance risks.

A further point of consideration in our research concerns the nature of the intergroup attitudes being measured, where in Study 1 we evaluated attitudes toward immigrants and in Study 2 attitudes toward Mexican Americans rather than attitudes toward other potential groups that could have been used. We chose target groups that have experienced disadvantage within the United States (e.g., Immigrants; Mexican Americans).

While we could have chosen other groups that may experience bias from people endorsing individualizing foundations, those groups tend to be high status groups (CEOs, managers, lawyers, etc.) that do not experience systematic prejudice within society and thus are not as vulnerable as the groups we had selected. There also is a growing literature suggesting that Individualizing foundations relate to more positive intergroup attitudes and a broader group focus than do the binding foundations and this could be one explanation for our findings (Forsberg et al., 2019; Stewart and Morris, 2021).

Another interpretation of our findings that could be made is that the threat responses in our study may have gone through a process of moralization for those higher in the binding foundations (see the work of Hoover et al., 2021). This moralization process occurs when those higher in the binding foundations perceive threat as more self-justified due to a perception of the violation of their binding foundations, leading to higher perceived justifications of negatively held attitudes. Hoover et al. (2021) measured this concept using a variable called ‘perceived moral wrongness’ and suggested such processes can underlie strongly negative attitudes. This work also highlights an important distinction in moral foundations in regard to the variable of “extreme behavioral expressions of prejudice” (EBEP). Hoover et al. (2021) used an experimental manipulation to violate individualizing values or to violate binding values. They found that in the case of binding foundation violations, support for extreme behavioral expressions of prejudice (EBEP’s) was increased for those higher (vs. lower) in binding foundations and was mediated by a sense of perceived moral wrongness. A similar pattern was not observed for violation of individualizing foundations. This suggests that using groups more likely to violate individualizing values would be unlikely to provoke such strong negative attitudes for those high in the individualizing values, but using groups that violate binding foundations may prompt more negative attitudes. This would support our conclusion that those high in binding foundations were more prone to extremely negative responses and points to the need to further understand the role of binding values in relation to negative attitudes and strong ideologies.

Across our two studies, we observed that individualizing moral foundations were related to *less* negativity toward immigrants in Study 1 (*R*^2^ = 0.05), and to less negative intergroup attitudes (*R*^2^ = 0.09) and less negative-focused attitudes (*R*^2^ = 0.03) toward Mexican Americans in Study 2. The binding moral foundations were related to *more* negative attitudes in Study 1 (*R*^2^ = 0.14), and to more negative intergroup attitudes toward Mexican Americans (*R*^2^ = 0.05) and more negative-focused attitudes toward Mexican Americans in Study 2 (*R*^2^ = 0.11). Overall, most of the effects were medium effect sizes, which indicates that these measures were not demonstrating floor effects in which all participants were answering at the bottom of the scales; participants were also showing good variability in responses. In addition, there was an interesting finding with the negative-focused measure in which individualizing foundations were less strongly related to it (*R*^2^ = 0.03) than to the attitude measure that included positive and negative items (*R*^2^ = 0.09) while the binding foundations were more strongly related to the negative-focused measure (*R*^2^ = 0.11) than to the balanced measure (*R*^2^ = 0.05). This finding suggests that binding foundations may be more strongly related to very negative sentiments such as disapproval, hatred, and disdain, and future research could investigate this idea more thoroughly. This finding is in line with the research by Hoover et al. (2021) on extreme behavioral expressions of prejudice.

Researchers have sometimes questioned the order of influence between the moral foundations and ideologies, which has been difficult to establish (see McAdams et al., 2008; Koleva et al., 2012). There are theoretical debates indicating that basic personality dispositions, of which moral foundations could be included, may predict RWA and SDO given that RWA and SDO are social attitudes that may develop in late adolescence (see Sibley and Duckitt, 2013; also Altemeyer, 1998; Van Hiel et al., 2004). Additionally, moral foundations are hypothesized to have evolved as fundamental moral values (Haidt, 2012) and theory suggests that moral foundations are derived from rapid intuitive reactions that inform our social judgments and attitudes on social issues (see Haidt, 2001 for the SIM Model). In our studies, we used moral foundations as predictors of RWA and SDO, and RWA and SDO as mediators. We empirically tested this ordering with structural equation models and found that our ordering had better fit than models using RWA and SDO as predictors (see Supplementary materials: ‘Alternative Order Analysis,’ Supplementary Tables S.3–S.5). However, future research will need to confirm the direction of influence using manipulated variables or cross-lagged longitudinal designs.

Studies 1 and 2 also included participants from a variety of ethnic backgrounds, and one question that may be asked is the extent to which the findings are robust if we included only participants who identified as being white, or if we included only participants who had lived in the United States for a set number of years. Including only participants who were white did not change any of the general patterns observed between the individualizing and binding foundations and intergroup variables in Study 1 concerning perceptions of immigrants or Study 2 for Mexican Americans (see Supplementary materials: ‘Controlling for Other Factors’); it also did not change the results of the mediational analyses. Including only participants who had lived in the United States for 10 or more years as Richeson and Nussbaum (2004) had done also did not change any of the general patterns or significant results observed in Study 1 or 2 (removing zero participants from Study 1, and only one participant from Study 2 who was not born in the United States). We also explored alternative analyses and found that the binding foundations, and not individualizing foundations, were strongly and significantly related to more RWA. The SDO variable was also more strongly related to more investment in individualizing foundations than with the binding foundations, which gives confidence in our models (see Supplementary materials for alternative model); within the Supplementary materials we also considered comparisons using structural equation models on the full sample of participants for each study to support the construction of our models (see Supplementary materials: ‘Alternative Order Analysis,’ Supplementary Tables S.3–S.5). Conducting alternative analyses across all studies suggested that neither ethnicity nor years living in the country was an explanation of the effects observed. Finally, we conducted alternative modeling around serial mediation in which we tested the path from individualizing (or the binding) predictor to the mediator of threat and then from threat to the mediator SDO (or RWA) and finally to the attitudes outcome variable (for example: Binding ->Threat -> RWA -> Attitudes). The serial mediation path in which threat always preceded the RWA or SDO ideology variable was not significant in each model whereas the path from the predictor to threat to outcome remained significant in every case; these results supported our parallel mediations used in the main analyses (see Supplementary materials: ‘Serial Mediation Analyses,’ Supplementary Figures S.20–S.25). We then conducted alternative serial mediation models reversing the order of the two mediators in the serial mediation design so that the social ideology mediator (RWA/SDO) always preceded the threat mediator (for example: Binding -> RWA -> Threat -> Attitudes). We found that, in each case, serial mediation was successful. However, in about half of cases, the threat mediation path also remained significant. Overall, this serial mediation may suggest that moral foundations influence the RWA and SDO ideologies which in turn may be leading to increased threat perceptions as a mechanism of effect. Future research will need to examine these findings further to confirm this idea (see Supplementary materials: ‘Serial Mediation Analyses with Alternative Mediator Order,’ Supplementary Figures S.26–S.31). We also conducted models including both the subcomponents of threat (realistic and symbolic) entered as mediators, and we observed that both types of threat were significant mediators and continued to cancel out the effects of RWA and SDO across models (see Supplementary materials: ‘Parallel Multiple-Mediation Analyses with Realistic and Symbolic Threat,’ Supplementary Figures S.14–S.19).

While our research has detected relationships between moral values, threat, and strong ideologies, it is worth noting one limitation of the measurement of RWA and SDO in the current research. The average scores on the seven-point Likert response scales for the RWA and SDO measures were fairly low across our two studies. This may be partially expected due to the strength of attitudes being measured when considering that these strong social ideologies are often linked to highly negative intergroup attitudes. For example, the SDO measure (Pratto et al., 1994) considers attitudes such as stepping on other groups, which we would not expect to be endorsed by those with moderate views. Perhaps we have detected fairly low averages on these measures because of the extremeness of the views in those measures; our observed scores are consistent with work by other researchers such as Koleva et al. (2012) who detected similarly low average values for both RWA and SDO across two studies. This is a limitation of our measurement in a general population, which may limit the inferences we can make about people who endorse RWA and SDO most strongly. We, however, would expect that the effects observed from those with strong endorsements would be even larger than the effects we observed. Our study may therefore be a better reflection of the low to moderate levels of these RWA and SDO values in a general population, which still have important implications for intergroup relations as demonstrated by the consistent patterns detected. Future research will need to examine these ideas.

It is also worth considering how future versions of MFT may improve the ability of researchers to understand the relationships between sets of values and intergroup variables. Haidt (2012) originally noted that the fairness foundation may be broadly defined in its current state, rather than more specifically. For example, those higher in binding foundations may be more prone to endorse notions of fairness that have a strong focus around proportionality where this definition is primarily oriented around contribution levels in deciding fairness evaluations. In contrast, those high in the individualizing foundations may be more supportive of an equality and welfare focus as being important in fairness evaluations. Forthcoming research by Atari et al. (2022) has measured and validated a distinction between proportionality and equality (see also Rai and Fiske, 2011) and will update the moral foundations questionnaire into the new MFQ-2 by dividing the fairness foundation into ‘equality’ and ‘proportionality’ foundations. The MFQ-2 also uses newly developed items to measure the moral foundations and has accounted for non-western nations in testing and development.

For example, one study using the MFQ-2 has analyzed data from 19 different nations and has indicated a more accurate ability to measure moral values from countries that are not western, educated, industrialized, rich, and democratic (see Henrich et al., 2010 on W.E.I.R.D countries; Atari et al., 2022). The MFQ-2 has also demonstrated a better ability to explain variance in relevant outcome measures as compared to the original MFQ, while further demonstrating high reliability. This early research has indicated that equality and proportionality tend not to be correlated especially in more western nations and are distinct moral foundations. While this is the case, both still correlate positively with the original fairness foundation and distinguish between different aspects of fairness. This new distinction in the MFQ-2 will provide an opportunity for future research to gain a more fine-grained analysis of how foundations relate to intergroup variables and improve the ability of researchers to more accurately measure moral values across cultures (Atari et al., 2022).

If immigrant groups and other ethnic minority groups, who might be perceived as immigrants, even if they are not, are being perceived as threatening by those high in binding foundations, this has important implications for intergroup relations. This is especially important given the likelihood of continuing immigration within the United States and around the world due to the need to maintain population and economic growth. Finding ways to reduce threat perceptions and negative attitudes directed toward ethnic minorities and immigrant groups may provide an important step in reducing intergroup and ideological-based tensions. In our analyses, we observed that accounting for threat perceptions in the multiple-mediation models significantly reduced the indirect effect of the individualizing to intergroup attitudes relationship through SDO, and also significantly reduced the indirect effect of the binding to attitudes relationship through RWA. This pattern of results supports the idea that threat processing may play a key role in both RWA and SDO, which is in-line with research on RWA (Stenner, 2005; Duckitt and Sibley, 2009; Van Leeuwen and Park, 2009; Federico et al., 2013) and on SDO (Morrison and Ybarra, 2008; Vezzali and Giovannini, 2010; Uenal, 2016), and also moral foundations (Van Leeuwen and Park, 2009; Van de Vyver et al., 2016; Sinn and Hayes, 2017). Thus, reducing perceived symbolic and realistic threats relating to harm and fairness, and relating to binding values of loyalty, authority, and purity may be avenues for reducing negative attitudes toward immigrants or other ethnic minorities in increasingly hostile contexts, and for improving dialogues between groups with strong ideologies. Study 2 further demonstrated that the patterns detected regarding attitudes toward immigrants in Study 1 also held when considering attitudes toward a different ethnic minority group (Mexican Americans). These findings suggest that the models presented here may also be useful for understanding moral foundations, threat perceptions, and strong social ideologies in the context of race-based attitudes more generally as well as immigration-focused attitudes.

Overall, the current research suggests some early steps that may aid our understanding of moral beliefs and intergroup relations, and ways to improve perceptions. Patterns of moral beliefs and strong ideologies may contribute to perceptions of immigrant groups and possibly many other ethnic-minority groups as being threatening and negative. Endorsement of the individualizing foundations of harm and fairness was related to less negative attitudes and less perceived threat, whereas endorsement of the binding foundations of loyalty, authority, and purity was related to more negative attitudes and more threat. The binding foundations to attitudes relationship was mediated by RWA, and the individualizing foundations to attitudes relationship was mediated by SDO; this effect occurred in Study 1 with attitudes toward immigrants and also in Study 2 with attitudes toward Mexican Americans, who are predominantly not immigrants (Noe-Bustamante et al., 2019; U.S. Census Bureau, 2019). The addition of threat to these mediation models eliminated mediational effects of RWA and SDO ideologies for moral foundations to attitudes relationships. Therefore, reducing such perceptions of perceived threat may play an essential role in reducing intergroup tensions. During times that emphasize threats in the United States and in Europe, this perception may exacerbate these tendencies for those who wish to avoid risk and threats. While this may be related to intergroup tensions, it may also provide an avenue to discuss ways to continue being an open and democratic society while protecting against threats.

## Data availability statement

The original contributions presented in this study are included in the article/Supplementary material, further inquiries can be directed to the corresponding author.

## Ethics statement

The studies involving human participants were reviewed and approved by the STEM Ethical Review Committee at the University of Birmingham and following British Psychological Society (BPS) and American Psychological Association (APA) ethical guidelines. The patients/participants provided their written informed consent to participate in this study.

## Author contributions

The study idea and design was developed by DM. Material in the studies was designed by DM and BS. Design of materials was prepared by DM with assistance from BS. Analysis of data was conducted by DM and BS. Writing up of studies was done by DM with assistance and revision from BS. Both authors contributed to the article and approved the submitted version.

## Footnotes

1 Additionally, we examine how the moral foundations relate to the perspective taking subscale of the interpersonal reactivity index (IRI: Davis, 1983). Previous research has not found a consistent difference in perspective taking by political ideology (Jost et al., 2003; Falk et al., 2012). However, some work in moral foundations has suggested that harm and fairness positively relates to perspective taking (Glenn et al., 2009) and individualizing foundations tend to be endorsed across the political spectrum (Haidt, 2007).

2 We conducted multiple mediation analyses to examine the influence of realistic and symbolic threat when realistic and symbolic threat were both entered as mediators in parallel. In each analysis, we found that both realistic and symbolic threat mediated the relationships between moral foundations and intergroup attitudes and the relationships between moral foundations and negative-focused attitudes when the other mediator was present in the model. As was the case with the combined threat variable, RWA and SDO were not significant mediators in any of the mediational analyses when realistic and symbolic threat were also included as mediators. This supports our use of a combined threat measure within the research (see Supplementary materials: ‘Parallel Multiple-Mediation Analyses with Realistic and Symbolic Threat’ of the Supplementary Figures S.14–S.19).

## Supplementary material

Supplementary Table 1 Supplementary materials.

Supplementary Table 2 Data dictionary for Study 1.

Supplementary Table 3 Data dictionary for Study 2.

Supplementary Table 4 Dataset for Study 1.

Supplementary Table 5 Dataset for Study 2.

Supplementary Table 6 Models using negative focused attitudes as the outcome variable, Study 2 (*N* = 388).
