# Supplementary material for: Moral values, social ideologies and threat-based cognition: Implications for intergroup relations
Source: Front Psychol. 2022 Oct 6;13:869121. doi: 10.3389/fpsyg.2022.869121 (PMC9582249; doi:10.3389/fpsyg.2022.869121)
Supplement: Supplementary Table 1 — Supplementary materials. [file Table_1.DOCX]

**Table 1: Supplemental Analyses for Morris & Stewart.**

All analyses in the supplemental materials are conducted on samples without participants who failed the MFQ attention checks.

**Table S.1: Study 1 Correlation Matrix for Study Variables (N = 157)**

|  | Ind | Bind | Harm | Fair | Loy | Auth | Pur | PT | SDO | RWA | Atts | Thr |
| --- | --- | --- | --- | --- | --- | --- | --- | --- | --- | --- | --- | --- |
| Indiv | 1 |  |  |  |  |  |  |  |  |  |  |  |
| Binding | .082 | 1 |  |  |  |  |  |  |  |  |  |  |
| Harm | .885*** | .162* | 1 |  |  |  |  |  |  |  |  |  |
| Fairness | .849*** | -.032 | .505*** | 1 |  |  |  |  |  |  |  |  |
| Loyalty | .108 | .848*** | .170* | .006 | 1 |  |  |  |  |  |  |  |
| Authority | .067 | .881*** | .123 | -.015 | .699*** | 1 |  |  |  |  |  |  |
| Purity | .050 | .897*** | .137 | -.062 | .598*** | .670*** | 1 |  |  |  |  |  |
| PT | .261*** | -.018 | .200* | .258** | .081 | -.041 | -.065 | 1 |  |  |  |  |
| SDO | -.657*** | .296*** | -.508*** | -.641*** | .225** | .267*** | .280*** | -.355*** | 1 |  |  |  |
| RWA | -.143 | .757*** | -.002 | -.263*** | .571*** | .624*** | .757*** | -.108 | .436*** | 1 |  |  |
| Attitudes | -.233** | .369*** | -.177* | -.231** | .268*** | .322*** | .364*** | -.332*** | .503*** | .449*** | 1 |  |
| Threat | -.241** | .499*** | -.141 | -.287*** | .431*** | .444*** | .439*** | -.269*** | .562*** | .578*** | .791*** | 1 |

**p <* .05, ** *p <* .01, ****p* < .001

**Table S.2: Study 2 Correlation Matrix for Study Variables (N = 388)**

|  | Indiv | Binding | Harm | Fairness | Loyalty | Author | Purity | SDO | RWA | Attitude | N. Att | Threat |
| --- | --- | --- | --- | --- | --- | --- | --- | --- | --- | --- | --- | --- |
| Indiv | 1 |  |  |  |  |  |  |  |  |  |  |  |
| Binding | .037 | 1 |  |  |  |  |  |  |  |  |  |  |
| Harm | .877*** | .101* | 1 |  |  |  |  |  |  |  |  |  |
| Fairness | .859*** | -.040 | .506*** | 1 |  |  |  |  |  |  |  |  |
| Loyalty | .003 | .786*** | .052 | -.050 | 1 |  |  |  |  |  |  |  |
| Authority | -.012 | .892*** | .049 | -.074 | .639*** | 1 |  |  |  |  |  |  |
| Purity | .084 | .885*** | .137** | .005 | .488*** | .682*** | 1 |  |  |  |  |  |
| SDO | -.522*** | .357*** | -.409*** | -.501*** | .318*** | .360*** | .261*** | 1 |  |  |  |  |
| RWA | -.167*** | .732*** | -.097 | -.196*** | .436*** | .640*** | .750*** | .442*** | 1 |  |  |  |
| Attitudes | -.292*** | .216*** | -.260*** | -.247*** | .098 | .206*** | .228*** | .455*** | .322*** | 1 |  |  |
| Neg. Att | -.165** | .326*** | -.105* | -.184*** | .209*** | .291*** | .320*** | .414*** | .361*** | .626*** | 1 |  |
| Threat | -.261*** | .516*** | -.207*** | -.247*** | .348*** | .503*** | .463*** | .584*** | .603*** | .597*** | .606*** | 1 |

**p <* .05, ** *p <* .01, ****p* < .001

**Study 1: Alternative Order Analysis**

Some researchers have suggested that the social-political attitudes of RWA and SDO may be exogenous and predict moral foundations (Federico et al., 2013) instead of moral foundations predicting RWA and SDO (Sibley & Duckitt, 2013). Given that we have used cross-sectional data, this theoretical debate about order is also an empirical question. We can use structural equation modeling to check whether the model of moral foundations as predictors demonstrates better fit than the model of RWA and SDO as predictors. There, however, is one important problem with using structural equation modeling with mediation models with just one mediator (i.e., tri-variate mediation model problem). Research has shown that we cannot test for differences in fit between models by reversing the directed arrows within the mediation model so that Individualizing mediates the SDO to Attitudes relationship instead of SDO mediating the Individualizing to Attitudes relationship as in Figure 1; we cannot make these comparisons because they are of the same equivalence class, and thus, these reversed models produce identical fit statistics to each other (MacCallum, Wegener, Uchino, & Fabrigar, 1993; Thoemmes, 2015). We, however, can use models in which both RWA and SDO are included in order to compare the MFQ as predictor models to the RWA and SDO as predictors models.

Therefore, we compared a model in which the Individualizing to Attitudes relationship was mediated by SDO and RWA (similar to Fig. 1 of the manuscript) to an Alternative model in which SDO and RWA predicted Attitudes and was mediated by the Individualizing foundation. We also compared models in which the Individualizing foundation was replaced with Binding foundation, and we used the Akaike Information Criterion (AIC) and the Bayesian Information Criterion (BIC) to compare fit. These fit indices are necessary when comparing the non-nested models that we are comparing, and lower AIC and BIC values indicate better fit (Kline, 1998). If social-political attitudes are exogenous to moral foundations, then those models would have lower AIC and BIC values. Table S.3 demonstrates that contrary to the socio-political as exogenous hypothesis, both Moral Foundations as Predictor Models fit better than the Alternative Models. These results support our use of moral foundations as predictors.

**Table S.3**

*Information criteria comparing the Moral Foundations Predictor Models to the SDO and RWA Alternative Models.*

| Fit Indices | Model | |
| --- | --- | --- |
|  | Individualizing as Predictor | Alternative |
|  |  |  |
| AIC | 2655.13 | 2664.19 |
| BIC | 2963.81 | 2972.87 |
|  |  |  |
|  | Binding as Predictor | Alternative |
| AIC | 3276.93 | 3294.63 |
| BIC | 3622.28 | 3639.99 |

**Study 2: Alternative Order Analysis**

In Study 2, following the methods outlined in Study 1, we also produced Structural Equation models (SEM) to justify the use of the moral foundations as predictor variables for the intergroup attitudes models reported (See Table S.4). In Study 2 we replicated the SEM findings from Study 1 in which the models with Individualizing and Binding as predictors had better fit than those with RWA and SDO as predictors as opposed to mediators.

**Table S.4**

*Information criteria comparing the Moral Foundations Predictor Models to the SDO and RWA Alternative Models for the Intergroup Attitudes outcome measure.*

| Fit Indices | Model | |
| --- | --- | --- |
|  | Individualizing as Predictor | Alternative |
|  |  |  |
| AIC | 3622.42 | 3644.30 |
| BIC | 4022.48 | 4044.36 |
|  |  |  |
|  | Binding as Predictor | Alternative |
| AIC | 4494.52 | 4554.46 |
| BIC | 4942.11 | 5002.05 |

**Study 2: Negative-Focused Attitudes Alternative Order Analysis**

In Study 2 following the methods outlined in Study 1, we also produced Structural Equation models to justify the use of the moral foundations as predictor variables for the negative-focused attitudes models reported (See Table S.5). Once again, the models with Individualizing and Binding as predictors had better fit than the alternative models with SDO and RWA as predictors.

**Table S.5**

*Information criteria comparing the Moral Foundations Predictor Models to the SDO and RWA Alternative Models for the Negative-Focused Attitudes outcome measure.*

| Fit Indices | Model | |
| --- | --- | --- |
|  | Individualizing as Predictor | Alternative |
|  |  |  |
| AIC | 3686.99 | 3708.81  7 |
| BIC | 4087.05 | 4108.87 |
|  |  |  |
|  | Binding as Predictor | Alternative |
| AIC | 4545.63 | 4605.59 |
| BIC | 4993.23 | 5053.18 |

Raftery (1995) noted that a difference of more than 10 points in BIC levels between two models is associated with “posterior odds of about 150:1” (p.139). In Table S.3, we observe a difference of 9.06, which is termed a “strong” difference, *p* < .01, while our other model differences (Table’s S.4 and S.5) exceed this threshold qualifying as “very strong”, *p* < .001. This lends support for our ordering in our models. Burnham and Anderson (2002) similarly suggest that when a model has a difference of 4 to 7 points in AIC there is “considerably less” evidence for the higher AIC model. Our models also exceed these thresholds, again lending support to our choices around ordering.

**Controlling For Other Factors**

Study 1: Effects of Moral Foundations on Intergroup Attitudes Controlling for Participant Race and Years Lived in the U.S.

We filtered non-white participants from the sample in order to control for the influence of race on the analyses. We then conducted regression models examining the influence of the moral foundations on Intergroup Attitudes and Threat (N = 123 across models). To examine Intergroup Attitudes a linear regression was conducted on this filtered sample entering the Individualizing foundation score as a predictor and Intergroup Attitudes as the outcome variable. It was found that the Individualizing foundations were still a significant and negative predictor of Intergroup Attitudes (Model *R^2^* = .07, *ϐ* = -.27, *t* = -3.03, *p* = .003) in which more Individualizing endorsement was related to less negative attitudes. Conducting a linear regression in which the Binding foundation score acted as a predictor found that Binding foundation score was still a positive and significant predictor of Intergroup Attitudes (Model *R^2^* = .12, *ϐ* = .35, *t* = 4.08, *p* < .001), in which, more binding endorsement related to more negative attitudes.

To control for the influence of Years Lived in the U.S. models were constructed (N =157) entering this variable alongside the different sets of moral foundations. In the presence of the Years Lived in the U.S. variable the Individualizing foundation score was still a significant and negative predictor of Intergroup Attitudes (Model *R^2^* = .08, Individualizing Foundations predictor: *ϐ* = -.24, *t* = -3.09, *p* = .002) with more Individualizing endorsement being related to less negative attitudes. Running the same model entering the Binding foundation score alongside years lived in the U.S again found that Binding foundations were a significant and positive predictor of Intergroup Attitudes when controlling for Years Lived in the U.S. (Model *R^2^* = .15, Binding Foundations predictor: *ϐ* = .36, *t* = 4.77, *p* < .001), with more endorsement being related to more negative attitudes.

Study 1: Effects of Moral Foundations on Threat Controlling Participant Race and Years Lived in the U.S.

We filtered non-white participants from the sample in order to control for the influence of race on the analyses (N = 123). Again, as in the case of the Intergroup Attitudes analysis, this did not change the findings as Individualizing foundation score negatively and significantly predicted Perceived Threat (Model *R^2^* = .06, *ϐ* = -.25, *t* = -2.82, *p* = .006), while the Binding foundation score positively and significantly predicted Perceived Threat (Model *R^2^* = .28, *ϐ* = .53, *t* = 6.79, *p* < .001).

To control for the influence of Years Lived in the U.S. models (N= 157) were constructed entering this variable alongside the moral foundations as predictors. In the presence of the Years Lived in the U.S. variable the Individualizing foundation score was still a significant and negative predictor of Perceived Threat (Model *R^2^* = .10, Individualizing Foundations predictor: *ϐ* = -.25, *t* = -3.26, *p* = .001). Running the same model entering the Binding foundation score alongside years lived in the U.S again found that Binding foundations were a significant and positive predictor of Perceived Threat when controlling for Years Lived in the U.S. (Model *R^2^* = .27, Binding Foundations predictor: *ϐ* = .48, *t* = 6.98, *p* < .001).

**Study 1: Mediation Models**

Conducting the four mediation models while using the sample including participants who were white and who passed the MFQ attention checks did not change any of the general findings of the four mediation models (see below);

**FIGURE S.1:** *Study 1: Controlling for the influence of race (N = 123) Individualizing to Intergroup Attitudes mediated by SDO.*

Individualizing

Foundations

SDO

*b* = -1.20, *p* < .001

*b* = .79, *p* < .001

Total effect, *b* = -.63, *p* = .003

Direct effect, *b* = .31, *p* = .220

Indirect effect, *b* = -.94, 95% CI [-1.50, -.50], *CSIE* = -.40, CI [-.63, -.21]

Intergroup Attitudes

**FIGURE S.2:** *Study 1: Controlling for the influence of race (N = 123) Binding to Intergroup Attitudes mediated by RWA.*

Binding

Foundations

RWA

*b* = .96, *p* < .001

*b* = .72, *p* < .001

Total effect, *b* = .60, *p* < .001

Direct effect, *b* = -.09, *p* = .691

Indirect effect, *b* = .69, 95% CI [.37, 1.04], *CSIE* = .40, CI [.22, .58]

Intergroup Attitudes

**FIGURE S.3:** *Study 1: Controlling for the influence of race (N = 123) Individualizing to Intergroup Attitudes mediated by Threat with SDO in the model.*

Individualizing

Foundations

SDO

*b* = -1.20, *p* < .001

*b* = -.04, *p* = .718

Total effect, *b* = -.63, *p* = .003

Direct effect, *b* = -.21, *p* = .246

SDO indirect effect, *b* = .05, 95% CI [-.28, .31], *CSIE* = .02, CI [-.12, .14]

Threat indirect effect, *b* = -.47, 95% CI [-.81, -.10], *CSIE* = -.20, CI [-.34, -.03]

Intergroup Attitudes

Threat

*b* = -.51, *p* = .006

*b* = .93, *p* < .001

**FIGURE S.4:** *Study 1: Controlling for the influence of race (N = 123) Binding to Intergroup Attitudes mediated by Threat with RWA in the model.*

Binding Foundations

RWA

*b* = .96, *p* < .001

*b* = .01, *p* = .927

Total effect, *b* = .60, *p* < .001

Direct effect, *b* = -.19, *p* = .188

RWA indirect effect, *b* = .01, 95% CI [-.25, .28], *CSIE* = .01, CI [-.14, .16]

Threat indirect effect, *b* = .79, 95% CI [.52, 1.09], *CSIE* = .45, CI [.31, .61]

Intergroup Attitudes

Threat

*b* = .79, *p* < .001

*b* = 1.00, *p* < .001

**FIGURE S.5:** *Alternative Model: Binding to Intergroup Attitudes mediated by SDO (N = 157).*

Binding

Foundations

SDO

*b* = .38, *p* < .001

*b* = .61, *p* < .001

Total effect, *b* = .67, *p* < .001

Direct effect, *b* = .44, *p* = .001

Indirect effect, *b* = .23, 95% CI [.07, .47], *CSIE* = .13, CI [.04, .24]

Intergroup Attitudes

**Study 2**

**Table S.6:** *Orders of Counterbalanced Materials as Administered to Participants in Study 2* (*Note:* ‘Attitudes’ included the Intergroup Attitudes measure followed by Negative Attitudes measure in each case).

| Order | Measures | | | | | | | |
| --- | --- | --- | --- | --- | --- | --- | --- | --- |
| 1 | MFQ | Filler 1 | RWA | SDO | FILLER 2 | ATTITUDES | THREAT | P.TAKING |
| 2 | MFQ | Filler 1 | RWA | SDO | FILLER 2 | ATTITUDES | P.TAKING | THREAT |
| 3 | MFQ | Filler 1 | RWA | SDO | FILLER 2 | THREAT | ATTITUDES | P.TAKING |
| 4 | MFQ | Filler 1 | RWA | SDO | FILLER 2 | THREAT | P.TAKING | ATTITUDES |
| 5 | MFQ | Filler 1 | RWA | SDO | FILLER 2 | P.TAKING | ATTITUDES | THREAT |
| 6 | MFQ | Filler 1 | RWA | SDO | FILLER 2 | P.TAKING | THREAT | ATTITUDES |
| 7 | MFQ | Filler 1 | SDO | RWA | FILLER 2 | ATTITUDES | THREAT | P.TAKING |
| 8 | MFQ | Filler 1 | SDO | RWA | FILLER 2 | ATTITUDES | P.TAKING | THREAT |
| 9 | MFQ | Filler 1 | SDO | RWA | FILLER 2 | THREAT | ATTITUDES | P.TAKING |
| 10 | MFQ | Filler 1 | SDO | RWA | FILLER 2 | THREAT | P.TAKING | ATTITUDES |
| 11 | MFQ | Filler 1 | SDO | RWA | FILLER 2 | P.TAKING | ATTITUDES | THREAT |
| 12 | MFQ | Filler 1 | SDO | RWA | FILLER 2 | P.TAKING | THREAT | ATTITUDES |

**Figure S.6**: *Study 2: Controlling for the influence of Race and being Born in the U.S. (N = 298) Individualizing to Intergroup Attitudes Mediated by SDO.*

Individualizing

Foundations

SDO

*b* = -1.01, *p* < .001

*b* = .55, *p* < .001

Total effect, *b* = -.77, *p* < .001

Direct effect, *b* = -.21, *p* = .142

Indirect effect, *b* = -.56, 95% CI [-.76, -.39], *CSIE* = -.24, CI [-.32, -.17]

Intergroup Attitudes

**Figure S.7**: *Study 2: Controlling for the influence of Race and being Born in the US (N = 298) Binding to Intergroup Attitudes Mediated by RWA.*

Binding

Foundations

RWA

*b* = .99, *p* < .001

*b* = .47, *p* < .001

Total effect, *b* = .44, *p* < .001

Direct effect, *b* = -.03, *p* = .842

Indirect effect, *b* = .47, 95% CI [.27, .69], *CSIE* = .27, CI [.15, .38]

Intergroup Attitudes

**FIGURE S.8:** *Study 2: Controlling for the influence of Race and being Born in the US (N = 298) Individualizing to Intergroup Attitudes mediated by Threat with SDO in the model.*

Individualizing

Foundations

SDO

*b* = -1.01, *p* < .001

*b* = .13, *p* = .100

Total effect, *b* = -.77, *p* < .001

Direct effect, *b* = -.27, *p* = .035

SDO indirect effect, *b* = -.14, 95% CI [-.32, .03], *CSIE* = -.06, CI [-.14, .01]

Threat indirect effect, *b* = -.37, 95% CI [-.56, -.22], *CSIE* = -.16, CI [-.23, -.09]

Intergroup Attitudes

Threat

*b* = -.65, *p* < .001

*b* = .56, *p* < .001

**FIGURE S.9:** *Study 2: Controlling for the influence of Race and being Born in the US (N = 298) Binding to Intergroup Attitudes mediated by Threat with RWA in the model.*

Binding Foundations

RWA

*b* = .99, *p* < .001

*b* = .01, *p* = .928

Total effect, *b* = .44, *p* < .001

Direct effect, *b* = -.17, *p* = .159

RWA indirect effect, *b* = .01, 95% CI [-.15, .17], *CSIE* = .005, CI [-.09, .10]

Threat indirect effect, *b* = .60, 95% CI [.45, .79], *CSIE* = .35, CI [.27, .44]

Intergroup Attitudes

Threat

*b* = .85, *p* < .001

*b* = .71, *p* < .001

**Figure S.10**: *Study 2: Controlling for the influence of Race and being Born in the US (N = 298) Individualizing to Negative-Focused Attitudes Mediated by SDO.*

Individualizing

Foundations

SDO

*b* = -1.01, *p* < .001

*b* = .73, *p* < .001

Total effect, *b* = -.50, *p* = .002

Direct effect, *b* = .24, *p* = .182

Indirect effect, *b* = -.74, 95% CI [-1.06, -.49], *CSIE* = -.26, CI [-.36, -.18]

Negative Attitudes

**Figure S.11**: *Study 2: Controlling for the influence of Race and being Born in the US (N = 298) Binding to Negative-Focused Attitudes Mediated by RWA.*

Binding

Foundations

RWA

*b* = .99, *p* < .001

*b* = .44, *p* < .001

Total effect, *b* = .67, *p* < .001

Direct effect, *b* = .23, *p* = .163

Indirect effect, *b* = .44, 95% CI [.20, .71], *CSIE* = .21, CI [.10, .32]

Negative Attitudes

**FIGURE S.12:** *Study 2: Controlling for the influence of Race and being Born in the US (N = 298) Individualizing to Negative-Focused Attitudes mediated by Threat with SDO in the model.*

Individualizing

Foundations

SDO

*b* = -1.01, *p* < .001

*b* = .23, *p* = .026

Total effect, *b* = -.50, *p* = .002

Direct effect, *b* = .17, *p* = .283

SDO indirect effect, *b* = -.23, 95% CI [-.51, .02], *CSIE* = -.08, CI [-.17, .01]

Threat indirect effect, *b* = -.44, 95% CI [-.71, -.25], *CSIE* = -.16, CI [-.24, -.09]

Negative Attitudes

Threat

*b* = -.65, *p* < .001

*b* = .68, *p* < .001

**FIGURE S.13:** *Study 2: Controlling for the influence of Race and being Born in the US (N = 298) Binding to Negative-Focused Attitudes mediated by Threat with RWA in the model.*

Binding Foundations

RWA

*b* = .99, *p* < .001

*b* = -.07, *p* = .569

Total effect, *b* = .67, *p* < .001

Direct effect, *b* = .08, *p* = .574

RWA indirect effect, *b* = -.07, 95% CI [-.29, .13], *CSIE* = -.03, CI [-.14, .07]

Threat indirect effect, *b* = .66, 95% CI [.44, .93], *CSIE* = .32, CI [.23, .41]

Negative Attitudes

Threat

*b* = .85, *p* < .001

*b* = .78, *p* < .001

**Parallel Multiple-Mediation Analyses with Realistic and Symbolic Threat**

We conducted multiple mediation analyses to examine the influence of Realistic and Symbolic Threat when Realistic and Symbolic Threat were both entered as mediators in parallel. In each analysis, we found that both Realistic and Symbolic Threat mediated the relationships between moral foundations and Intergroup Attitudes and Negative-Focused Attitudes when the other mediator was present in the model. As was the case with the combined Threat variable, RWA and SDO were not significant mediators in any of the mediational analyses. This supports our use of a combined Threat measure within the research.

**Figure S.14:** *Study 1: Multiple Mediation Model of Individualizing to Intergroup Attitudes Entering SDO, Symbolic Threat and Realistic Threat as Mediators (N = 157).*

*b =* .46*, p < .*001

*b* = .43, *p* < .001

*b* = -.46, *p* = .003

Individualizing

Foundations

SDO

*b* = -1.15, *p* < .001

*b* = .12, *p* = .269

Total effect, *b* = -.58, *p* = .003

Direct effect, *b* = .01, *p* = .958

SDO indirect effect, *b* = -.14, 95% CI [-.44, .12]

Symbolic Threat indirect effect, *b* = -.20, 95% CI [-.35, -.06]

Realistic Threat indirect effect, *b* = -.25, 95% CI [-.48, -.05]

Intergroup Attitudes

Symbolic

Threat

*b = -.53, p =* .004

Realistic

Threat

**Figure S.15:** *Study 1: Multiple Mediation Model of Binding to Intergroup Attitudes Entering RWA, Symbolic Threat and Realistic Threat as Mediators (N = 157).*

*b =* .49*, p < .*001

*b* = .48, *p* < .001

*b* = .72, *p* < .001

Binding

Foundations

RWA

*b* = .96, *p* < .001

*b* = .03, *p* = .785

Total effect, *b* = .67, *p* < .001

Direct effect, *b* = -.09, *p* = .514

RWA indirect effect, *b* = .03, 95% CI [-.20, .29]

Symbolic Threat indirect effect, *b* = .35, 95% CI [.16, .54]

Realistic Threat indirect effect, *b* = .38, 95% CI [.19, .61]

Intergroup Attitudes

Symbolic

Threat

*b = .*78*, p <* .001

Realistic

Threat

**Figure S.16:** *Study 2: Multiple Mediation Model of Individualizing to Intergroup Attitudes Entering SDO, Symbolic Threat and Realistic Threat as Mediators (N = 388).*

*b =* .26*, p < .*001

*b* = .30, *p* < .001

*b* = -.48, *p* < .001

Individualizing

Foundations

SDO

*b* = -.92, *p* < .001

*b* = .13, *p* = .071

Total effect, *b* = -.68, *p* < .001

Direct effect, *b* = -.25, *p* = .024

SDO indirect effect, *b* = -.12, 95% CI [-.27, .02]

Symbolic Threat indirect effect, *b* = -.14, 95% CI [-.24, -.06]

Realistic Threat indirect effect, *b* = -.16, 95% CI [-.29, -.07]

Intergroup Attitudes

Symbolic

Threat

*b = -.*63*, p <* .001

Realistic

Threat

**Figure S.17:** *Study 2: Multiple Mediation Model of Binding to Intergroup Attitudes Entering RWA, Symbolic Threat and Realistic Threat as Mediators (N = 388).*

*b =* .34*, p < .*001

*b* = .36, *p* < .001

*b* = .74, *p* < .001

Binding

Foundations

RWA

*b* = 1.00, *p* < .001

*b* = .05, *p* = .564

Total effect, *b* = .37, *p* < .001

Direct effect, *b* = -.25, *p* = .015

RWA indirect effect, *b* = .05, 95% CI [-.10, .21]

Symbolic Threat indirect effect, *b* = .27, 95% CI [.16, .39]

Realistic Threat indirect effect, *b* = .30, 95% CI [.18, .45]

Intergroup Attitudes

Symbolic

Threat

*b = .*88*, p <* .001

Realistic

Threat

**Figure S.18:** *Study 2: Multiple Mediation Model of Individualizing to Negative-Focused Attitudes Entering SDO, Symbolic Threat and Realistic Threat as Mediators (N = 388).*

*b =* .48*, p < .*001

*b* = .19, *p* = .029

*b* = -.48, *p* < .001

Individualizing

Foundations

SDO

*b* = -.92, *p* < .001

*b* = .17, *p* = .046

Total effect, *b* = -.44, *p* = .001

Direct effect, *b* = .10, *p* = .413

SDO indirect effect, *b* = -.16, 95% CI [-.37, .04]

Symbolic Threat indirect effect, *b* = -.09, 95% CI [-.19, -.01]

Realistic Threat indirect effect, *b* = -.30, 95% CI [-.48, -.14]

Negative Attitudes

Symbolic

Threat

*b = -.*63*, p <* .001

Realistic

Threat

**Figure S.19:** *Study 2: Multiple Mediation Model of Binding to Negative-Focused Attitudes Entering RWA, Symbolic Threat and Realistic Threat as Mediators (N = 388).*

*b =* .51*, p < .*001

*b* = .22, *p* = .012

*b* = .74, *p* < .001

Binding

Foundations

RWA

*b* = 1.00, *p* < .001

*b* = -.05, *p* = .615

Total effect, *b* = .65, *p* < .001

Direct effect, *b* = .07, *p* = .531

RWA indirect effect, *b* = -.05, 95% CI [-.22, .12]

Symbolic Threat indirect effect, *b* = .17, 95% CI [.06, .29]

Realistic Threat indirect effect, *b* = .45, 95% CI [.26, .68]

Negative Attitudes

Symbolic

Threat

*b = .*88*, p <* .001

Realistic

Threat

**Serial Mediation Analyses**

In order to test an alternative form of modelling, we conducted analyses testing serial-mediation in which mediators would be tested sequentially to examine whether the Threat to RWA/SDO path would mediate the relationships between moral foundations and the outcome variables (indirect effect via predictor to threat to social ideology variable to outcome). In each case we did not find support for serial mediation across the models in the studies. The Predictor to Threat to Attitudes indirect effect, however, remained significant in each model.

**Figure S.20:** *Study 1: (N = 157) A Model Testing Serial Mediation of the Relationship between Individualizing Foundations and Intergroup Attitudes Through Threat and SDO.*

*b* = -.97, *p* < .001

Individualizing

Foundations

Threat

*b* = -.50, *p* = .002

*b* = .89, *p* < .001

Total effect, *b* = -.58, *p* = .003

Direct effect, *b* = .01, *p* = .954

Indirect effect Indiv to Threat to Attitudes, *b* = -.44, 95% CI [-.74, -.13]

Indirect effect Indiv to SDO to Attitudes, *b* = -.12, 95% CI [-.38, .11]

Indirect effect Indiv to Threat to SDO to Attitudes, *b* = -.02, 95% CI [-.09, .02]

Intergroup Attitudes

SDO

*b* = .12, *p* = .261

*b = .*36*, p < .*001

**Figure S.21:** *Study 1: (N = 157) A Model Testing Serial Mediation of the Relationship between Binding Foundations and Intergroup Attitudes Through Threat and RWA.*

*b* = .79, *p* < .001

Binding

Foundations

Threat

*b* = .75, *p* < .001

*b* = .96, *p* < .001

Total effect, *b* = .67, *p* < .001

Direct effect, *b* = -.09, *p* = .527

Indirect effect Binding to Threat to Attitudes, *b* = .72, 95% CI [.49, .98]

Indirect effect Binding to RWA to Attitudes, *b* = .03, 95% CI [-.17, .22]

Indirect effect Binding to Threat to RWA to Attitudes, *b* = .01, 95% CI [-.04, .05]

Intergroup Attitudes

RWA

*b* = .03, *p* = .777

*b = .*23*, p < .*001

**Figure S.22:** *Study 2: (N = 388) A Model Testing Serial Mediation of the Relationship between Individualizing Foundations and Intergroup Attitudes Through Threat and SDO*

*b* = -.70, *p* < .001

Individualizing

Foundations

Threat

*b* = -.56, *p* < .001

*b* = .55, *p* < .001

Total effect, *b* = -.68, *p* < .001

Direct effect, *b* = -.25, *p* = .025

Indirect effect Indiv to Threat to Attitudes, *b* = -.31, 95% CI [-.46, -.18]

Indirect effect Indiv to SDO to Attitudes, *b* = -.09, 95% CI [-.20, .01]

Indirect effect Indiv to Threat to SDO to Attitudes, *b* = -.03, 95% CI [-.08, .004]

Intergroup Attitudes

SDO

*b* = .13, *p* = .070

*b = .*39*, p < .*001

**Figure S.23:** *Study 2: (N = 388) A Model Testing Serial Mediation of the Relationship between Binding Foundations and Intergroup Attitudes Through Threat and RWA*

*b* = .78, *p* < .001

Binding

Foundations

Threat

*b* = .82, *p* < .001

*b* = .70, *p* < .001

Total effect, *b* = .37, *p* < .001

Direct effect, *b* = -.25, *p* = .015

Indirect effect Binding to Threat to Attitudes, *b* = .57, 95% CI [.44, .72]

Indirect effect Binding to RWA to Attitudes, *b* = .04, 95% CI [-.08, .16]

Indirect effect Binding to Threat to RWA to Attitudes, *b* = .01, 95% CI [-.02, .04]

Intergroup Attitudes

RWA

*b* = .05, *p* = .565

*b = .*26*, p < .*001

**Figure S.24:** *Study 2: (N = 388) A Model Testing Serial Mediation of the Relationship between Individualizing Foundations and Negative-Focused Attitudes Through Threat and SDO*

*b* = -.70, *p* < .001

Individualizing

Foundations

Threat

*b* = -.56, *p* < .001

*b* = .69, *p* < .001

Total effect, *b* = -.44, *p* = .001

Direct effect, *b* = .10, *p* = .438

Indirect effect Indiv to Threat to Negative Attitudes, *b* = -.39, 95% CI [-.58, -.21]

Indirect effect Indiv to SDO to Negative Attitudes, *b* = -.12, 95% CI [-.28, .03]

Indirect effect Indiv to Threat to SDO to Negative Attitudes, *b* = -.04, 95% CI [-.10, .01]

Negative Attitudes

SDO

*b* = .17, *p* = .047

*b = .*39*, p < .*001

**Figure S.25:** *Study 2: (N = 388) A Model Testing Serial Mediation of the Relationship between Binding Foundations and Negative-Focused Attitudes Through Threat and RWA*

*b* = .78, *p* < .001

Binding

Foundations

Threat

*b* = .82, *p* < .001

*b* = .76, *p* < .001

Total effect, *b* = .65, *p* < .001

Direct effect, *b* = .07, *p* = .544

Indirect effect Bind to Threat to Negative Attitudes, *b* = .62, 95% CI [.44, .84]

Indirect effect Bind to RWA to Negative Attitudes, *b* = -.04, 95% CI [-.17, .09]

Indirect effect Bind to Threat to RWA to Negative Attitudes, *b* = -.01, 95% CI [-.05, .03]

Negative

Attitudes

RWA

*b* = -.05, *p* = .618

*b = .*26*, p < .*001

**Serial Mediation Analyses with Alternative Mediator Order**

In order to test an alternative form of serial mediation in which the order of the two mediators from the previous serial mediation models were reversed, we conducted analyses testing serial-mediation where in each case the social ideology mediator (RWA/SDO) always preceded the Threat mediator (serial mediation indirect effect via predictor to social ideology variable to threat to outcome). In each case, we now found support for serial mediation across the models in the studies. In around half of cases, the Threat path also remained significant. These models suggest that moral foundations are more likely to operate by influencing social ideology (RWA/SDO), which in turn increase threat perceptions that then increase negative outgroup attitudes. Future research will need to confirm this direction of effect using experimental methods.

**Figure S.26** *Study 1: (N = 157) A Model Testing Serial Mediation of the Relationship between Individualizing Foundations and Intergroup Attitudes Through SDO and Threat.*

*b* = .46, *p* = .010

Individualizing

Foundations

SDO

*b* = -1.15, *p* < .001

*b* = .12, *p* = .261

Total effect, *b* = -.58, *p* = .003

Direct effect, *b* = .01, *p* = .954

Indirect effect Indiv to Threat to Attitudes, *b* = .41, 95% CI [.10, .76]

Indirect effect Indiv to SDO to Attitudes, *b* = -.14, 95% CI [-.45, .11]

Indirect effect Indiv to SDO to Threat to Attitudes, *b* = -.86, 95% CI [-.1.16, -.57]

Intergroup Attitudes

Threat

*b* = .89, *p* < .001

*b = .*83*, p < .*001

**Figure S.27** *Study 1: (N = 157) A Model Testing Serial Mediation of the Relationship between Binding Foundations and Intergroup Attitudes Through RWA and Threat.*

*b* = .22, *p* = .150

Binding

Foundations

RWA

*b* = .96, *p* < .001

*b* = .03, *p* = .777

Total effect, *b* = .67, *p* < .001

Direct effect, *b* = -.09, *p* = .527

Indirect effect Bind to Threat to Attitudes, *b* = .21, 95% CI [-.07, .48]

Indirect effect Bind to RWA to Attitudes, *b* = .03, 95% CI [-.21, .27]

Indirect effect Bind to RWA to Threat to Attitudes, *b* = .51, 95% CI [.29, .78]

Intergroup Attitudes

Threat

*b* = .96, *p* < .001

*b = .*55*, p < .*001

**Figure S.28** *Study 2: (N = 388) A Model Testing Serial Mediation of the Relationship between Individualizing Foundations and Intergroup Attitudes Through SDO and Threat.*

*b* = .13, *p* = .212

Individualizing

Foundations

SDO

*b* = -.92, *p* < .001

*b* = .13, *p* < .070

Total effect, *b* = -.68, *p* < .001

Direct effect, *b* = -.25, *p* = .025

Indirect effect Indiv to Threat to Attitudes, *b* = .07, 95% CI [-.05, .20]

Indirect effect Indiv to SDO to Attitudes, *b* = -.12, 95% CI [-.27, .02]

Indirect effect Indiv to SDO to Threat to Attitudes, *b* = -.38, 95% CI [-.50, -.27]

Intergroup Attitudes

Threat

*b* = .55, *p* < .001

*b = .*75*, p < .*001

**Figure S.29** *Study 2: (N = 388) A Model Testing Serial Mediation of the Relationship between Binding Foundations and Intergroup Attitudes Through RWA and Threat.*

*b* = .25, *p* = .007

Binding

Foundations

RWA

*b* = 1.00, *p* < .001

*b* = .05, *p* = .565

Total effect, *b* = .37, *p* < .001

Direct effect, *b* = -.25, *p* = .015

Indirect effect Bind to Threat to Attitudes, *b* = .18, 95% CI [.04, .32]

Indirect effect Bind to RWA to Attitudes, *b* = .05, 95% CI [-.10, .20]

Indirect effect Bind to RWA to Threat to Attitudes, *b* = .40, 95% CI [.28, .52]

Intergroup Attitudes

Threat

*b* = .70, *p* < .001

*b = .*57*, p < .*001

**Figure S.30** *Study 2: (N = 388) A Model Testing Serial Mediation of the Relationship between Individualizing Foundations and Negative Attitudes Through SDO and Threat.*

*b* = .13, *p* = .212

Individualizing

Foundations

SDO

*b* = -.92, *p* < .001

*b* = .17, *p* = .047

Total effect, *b* = -.44, *p* = .001

Direct effect, *b* = .10, *p* = .438

Indirect effect Indiv to Threat to Negative Attitudes, *b* = .09, 95% CI [-.06, .25]

Indirect effect Indiv to SDO to Negative Attitudes, *b* = -.16, 95% CI [-.37, .04]

Indirect effect Indiv to SDO to Threat to Negative Attitudes, *b* = -.48, 95% CI [-.65, -.32]

Negative Attitudes

Threat

*b* = .69, *p* < .001

*b = .*75*, p < .*001

**Figure S.31** *Study 2: (N = 388) A Model Testing Serial Mediation of the Relationship between Binding Foundations and Negative Attitudes Through RWA and Threat.*

*b* = .25, *p* = .007

Binding

Foundations

RWA

*b* = 1.00, *p* < .001

*b* = -.05, *p* = .618

Total effect, *b* = .65, *p* < .001

Direct effect, *b* = .07, *p* = .544

Indirect effect Bind to Threat to Negative Attitudes, *b* = .19, 95% CI [.05, .35]

Indirect effect Bind to RWA to Negative Attitudes, *b* = -.05, 95% CI [-.22, .12]

Indirect effect Bind to RWA to Threat to Negative Attitudes, *b* = .43, 95% CI [.29, .60]

Negative Attitudes

Threat

*b* = .76, *p* < .001

*b = .*57*, p < .*001

**Testing Models with the Inclusion of General Political Orientation**

In order to examine the influence of political orientation, we included a variable measuring participant political orientation in each mediation model conducted across studies, including the negative attitudes models. A higher score on political orientation represented more liberalism (See demographics section of paper for details on the political orientation measure; Graham et al., 2009; Haidt, Graham, & Joseph, 2009).

These analyses would allow us to examine whether Threat still mediated the relationship between moral foundations and attitudes in the presence of a political orientation variable. The analyses further allowed us to demonstrate the distinctiveness of the more extreme SDO and RWA ideologies in contrast to the political orientation measure. Scholars such as Everett (2013) have emphasised the distinctiveness of RWA and SDO from political orientation. Such an analysis would be a particularly challenging test of our model.

Our findings generally support the idea that political orientation and RWA/SDO are distinct in their contribution to explaining variance in the basic models and that with the addition of Threat, the Threat variable generally explains variance over and above political orientation and SDO/RWA variables, showing its importance for understanding ideological cognition.

Study 1: Political Orientation Variable Mediation Analyses (N = 157)

We added the Political Orientation variable as a mediator across the models in Study 1. We report a summary of the findings below each model on the following pages;

[ANALYSES INCLUDED OVERLEAF]

**Figure S.32:** *Study 1: (N = 157) Testing the Inclusion of Political Orientation as a Mediator with SDO, Individualizing to Intergroup Attitudes Relationship.*

Individualizing

Foundations

SDO

*b* = -.1,15 *p* < .001

*b* = .68, *p* < .001

Total effect, *b* = -.58, *p* = .003

Direct effect, *b* = .50, *p* = .025

SDO indirect effect, *b* = -.78, 95% CI [-1.24, -.37]

Political indirect effect, *b* = -.29, 95% CI [-.51, -.10]

Intergroup Attitudes

Political

*b* = 1.27, *p* < .001

*b* = -.23, *p* = .001

We first conducted an analysis including Political Orientation as a Mediator alongside the SDO mediator in the Individualizing foundations to Intergroup Attitudes model.

Here SDO remained significant as a mediator. Political Orientation also acted as a significant mediator of the relationship.

**Figure S.33:** *Study 1: (N = 157) Testing the Inclusion of Political Orientation as a Mediator with SDO and Threat on the Individualizing to Intergroup Attitudes Relationship.*

*b =* -.04*, p = .*426

*b* = .87, *p* < .001

*b* = -.50, *p* = .002

Individualizing

Foundations

SDO

*b* = -1.15, *p* < .001

*b* = .11, *p* = .340

Total effect, *b* = -.58, *p* = .003

Direct effect, *b* = .03, *p* = .849

SDO indirect effect, *b* = -.12, 95% CI [-.44, .14]

Threat indirect effect, *b* = -.43, 95% CI [-.72, -.12]

Political indirect effect, *b* = -.05, 95% CI [-.19, .08]

Intergroup Attitudes

Threat

*b =* 1*.*27*, p <* .001

Political

We then conducted an analysis including Political Orientation as a mediator alongside the SDO and Threat mediators in the Individualizing foundations to Intergroup Attitudes model.

Here Threat was a significant mediator of the relationship, whereas Political Orientation and SDO did not significantly mediate this relationship.

**Figure S.34:** *Study 1: (N = 157) Testing the Inclusion of Political Orientation as a Mediator with RWA on the Binding to Intergroup Attitudes Relationship.*

Binding

Foundations

RWA

*b* = .96, *p* < .001

*b* = .33, *p* = .041

Total effect, *b* = .67, *p* < .001

Direct effect, *b* = .12, *p* = .523

RWA indirect effect, *b* = .32, 95% CI [-.003, .67]

Political indirect effect, *b* = .22, 95% CI [.07, .40]

Intergroup Attitudes

Political

*b* = -.89, *p* < .001

*b* = -.25, *p* < .001

We first conducted an analysis including Political Orientation as a Mediator alongside the RWA mediator in the Binding foundations to Intergroup Attitudes model.

Here Binding to attitudes was not mediated by RWA. While the *p*-value associated with the path from RWA to Intergroup Attitudes was less than .05, the indirect effect of RWA was not significant, though the confidence intervals indicate that this was close to attaining significance. Political orientation did mediate the relationship between Binding foundations and Intergroup Attitudes.

**Figure S.35:** *Study 1: (N = 157) Testing the Inclusion of Political Orientation as a Mediator with RWA and Threat on the Binding to Intergroup Attitudes Relationship.*

*b =* -.07*, p =* .176

*b* = .93, *p* < .001

*b* = .75, *p* < .001

Binding

Foundations

RWA

*b* = .96, *p* < .001

*b* = -.01, *p* = .920

Total effect, *b* = .67, *p* < .001

Direct effect, *b* = -.08, *p* = .559

RWA indirect effect, *b* = -.01, 95% CI [-.25, .25]

Threat indirect effect, *b* = .70, 95% CI [.46, .95]

Political indirect effect, *b* = .06, 95% CI [-.03, .17]

Intergroup Attitudes

Threat

*b = -.*89*, p <* .001

Political

We then conducted an analysis including Political Orientation as a mediator alongside the RWA and Threat mediators in the Binding foundations to Intergroup Attitudes model.

Here Threat was a significant mediator of the relationship, whereas Political Orientation and RWA did not significantly mediate this relationship.

Study 2: Political Orientation Variable Mediation Analyses (N = 388)

We conducted the same set of analyses for Study 2 adding the Political Orientation variable as a mediator across the models. We report a summary of the findings in text below each model;

**Figure S.36:** *Study 2: (N = 388) Testing the Inclusion of Political Orientation as a Mediator with SDO on the Individualizing to Intergroup Attitudes Relationship.*

In Study 2 we performed the same set of analyses in Study 1. We first conducted an analysis including Political Orientation as a Mediator alongside the SDO mediator in the Individualizing foundations to Intergroup Attitudes model.

Individualizing

Foundations

SDO

*b* = -.92, *p* < .001

*b* = .46, *p* < .001

Total effect, *b* = -.68, *p* < .001

Direct effect, *b* = -.17, *p* = .161

SDO indirect effect, *b* = -.42, 95% CI [-.59, -.27]

Political indirect effect, *b* = -.08, 95% CI [-.16, -.01]

Intergroup Attitudes

Political

*b* = .98, *p* < .001

*b* = -.08, *p* = .017

Here SDO remained significant as a mediator. Political Orientation also acted as a significant mediator of the relationship.

**Figure S.37:** *Study 2: (N = 388) Testing the Inclusion of Political Orientation as a Mediator with SDO and Threat on Individualizing to Intergroup Attitudes Relationships.*

*b =* .06*, p = .*073

*b* = .59, *p* < .001

*b* = -.56, *p* < .001

Individualizing

Foundations

SDO

*b* = -.92, *p* < .001

*b* = .16, *p* = .032

Total effect, *b* = -.68, *p* < .001

Direct effect, *b* = -.25, *p* = .020

SDO indirect effect, *b* = -.15, 95% CI [-.30, -.01]

Threat indirect effect, *b* = -.33, 95% CI [-.49, -.19]

Political indirect effect, *b* = .06, 95% CI [-.01, .13]

Intergroup Attitudes

Threat

*b = .*98*, p* < .001

Political

In Study 2 we then conducted an analysis including Political Orientation as a mediator alongside the SDO and Threat mediators in the Individualizing foundations to Intergroup Attitudes model.

Here Threat was a significant mediator of the relationship, however SDO was also a significant mediator (see upper confidence interval), the political indirect effect was not significant in this model.

**Figure S.38:** *Study 2: (N = 388) Testing the Inclusion of Political Orientation as a Mediator with RWA on Binding to Intergroup Attitudes Relationships.*

Binding

Foundations

RWA

*b* = 1.00, *p* < .001

*b* = .29, *p* = .004

Total effect, *b* = .37, *p* < .001

Direct effect, *b* = -.12, *p* = .308

RWA indirect effect, *b* = .29, 95% CI [.08, .49]

Political indirect effect, *b* = .20, 95% CI [.07, .34]

Intergroup Attitudes

Political

*b* = -1.50, *p* < .001

*b* = -.13, *p* = .002

In Study 2 we conducted an analysis including Political Orientation as a Mediator alongside the RWA mediator in the Binding foundations to Intergroup Attitudes model.

Here RWA remained significant as a mediator and Political Orientation also acted as a significant mediator of the relationship.

**Figure S.39:** *Study 2: (N = 388) Testing the Inclusion of Political Orientation as a Mediator with RWA and Threat on Binding to Intergroup Attitudes Relationships.*

*b =* -.00*, p =* .999

*b* = .70, *p* < .001

*b* = .82, *p* < .001

Binding

Foundations

RWA

*b* = 1.00, *p* < .001

*b* = .05, *p* = .595

Total effect, *b* = .37, *p* < .001

Direct effect, *b* = -.25, *p* = .016

RWA indirect effect, *b* = .05, 95% CI [-.12, .22]

Threat indirect effect, *b* = .57, 95% CI [.44, .73]

Political indirect effect, *b* = .00, 95% CI [-.13, .12]

Intergroup Attitudes

Threat

*b = -*1*.*50*, p <* .001

Political

We then conducted an analysis including Political Orientation as a mediator alongside the RWA and Threat mediators in the Binding foundations to Intergroup Attitudes model.

Here Threat was a significant mediator of the relationship whereas Political Orientation and RWA did not significantly mediate this relationship.

Study 2 Exploring the Impact of Political Orientation on the Negative Focused-Attitudes Models

We conducted the same set of analyses for Study 2 adding the Political Orientation variable as a mediator across the models which used the Negative-Focused Attitudes variable as an outcome measure. In each case we report a summary of the findings in text below each model;

**Figure S.40:** *Study 2: (N = 388) Testing the Inclusion of Political Orientation as a Mediator with SDO on Individualizing to Negative-Focused Attitudes Relationships.*

Individualizing

Foundations

SDO

*b* = -.92, *p* < .001

*b* = .58, *p* < .001

Total effect, *b* = -.44, *p* = .001

Direct effect, *b* = .19, *p* = .184

SDO indirect effect, *b* = -.53, 95% CI [-.79, -.31]

Political indirect effect, *b* = -.11, 95% CI [-.20, -.02]

Negative

Attitudes

Political

*b* = .98, *p* < .001

*b* = -.11, *p* = .008

We then conducted an analysis including Political Orientation as a mediator alongside the SDO mediator in the Individualizing foundations to Negative-Focused Attitudes model.

Here SDO acted as a mediator between Individualizing to Negative Attitudes. Political Orientation also acted as a significant mediator.

**Figure S.41:** *Study 2: (N = 388) Testing the Inclusion of Political Orientation as a Mediator, with SDO and Threat on Individualizing to Negative-Focused Attitudes Relationships.*

*b =* .07*, p* = *.*075

*b* = .74, *p* < .001

*b* = -.56, *p* < .001

Individualizing

Foundations

SDO

*b* = -.92, *p* < .001

*b* = .20, *p* = .020

Total effect, *b* = -.44, *p* = .001

Direct effect, *b* = .09, *p* = .481

SDO indirect effect, *b* = -.19, 95% CI [-.40, .01]

Threat indirect effect, *b* = -.41, 95% CI [-.63, -.23]

Political indirect effect, *b* = .07, 95% CI [-.02, .17]

Negative Attitudes

Threat

*b = .*98*, p <* .001

Political

In Study 2 we then conducted an analysis including Political Orientation as a mediator alongside the SDO and Threat mediators for the Individualizing foundations to Negative-Focused Attitudes model.

Here Threat was a significant mediator of the relationship between Individualizing and Negative-Focused Attitudes. SDO and Political Orientation were not significant mediators of this relationship.

**Figure S.42:** *Study 2: (N = 388) Testing the Inclusion of Political Orientation as a Mediator with RWA on Binding to Negative-Focused Attitudes Relationships.*

Binding

Foundations

RWA

*b* = 1.00, *p* < .001

*b* = .29, *p* = .014

Total effect, *b* = .65, *p* < .001

Direct effect, *b* = .23, *p* = .095

RWA indirect effect, *b* = .28, 95% CI [.05, .52]

Political indirect effect, *b* = .13, 95% CI [-.03, .31]

Negative

Attitudes

Political

*b* = -1.50, *p* < .001

*b* = -.09, *p* = .077

In Study 2 we conducted an analysis including Political Orientation as a Mediator alongside the RWA mediator in the Binding foundations to Negative-Focused Attitudes model.

Here RWA remained significant as a mediator, however Political Orientation was not a significant mediator of this relationship.

**Figure S.43:** *Study 2: (N = 388) Testing the Inclusion of Political Orientation as a Mediator with RWA and Threat on Binding to Negative-Focused Attitudes Relationships.*

*b =* .06*, p* = *.*146

*b* = .79, *p* < .001

*b* = .82, *p* < .001

Binding

Foundations

RWA

*b* = 1.00, *p* < .001

*b* = .01, *p* = .925

Total effect, *b* = .65, *p* < .001

Direct effect, *b* = .09, *p* = .456

RWA indirect effect, *b* = .01, 95% CI [-.19, .21]

Threat indirect effect, *b* = .65, 95% CI [.45, .87]

Political indirect effect, *b* = -.10, 95% CI [-.26, .06]

Negative Attitudes

Threat

*b = -*1*.*50*, p <* .001

Political

We then conducted an analysis including Political Orientation as a mediator alongside the RWA and Threat mediators in the Binding foundations to Negative-Focused Attitudes model.

Here Threat was a significant mediator of the relationship whereas Political Orientation and RWA did not significantly mediate this relationship.

**Supplemental References**

Burnham, K. P., & Anderson, D. R. (2002). *Model selection and multi-model inference: A practical information-theoretic approach* (2^nd^ ed.) New York*:* Springer.

Everett, J. A. C. (2013). The 12 item social and economic conservatism scale (SECS). *PloS one*, *8*(12), e82131.

Federico, C. M., Weber, C. R., Ergun, D., & Hunt, C. (2013). Mapping the connections between politics and morality: The multiple sociopolitical orientations involved in moral intuition. *Political Psychology*, *34*(4), 589-610.

Graham, J., Haidt, J., & Nosek, B. A. (2009). Liberals and conservatives rely on different sets of moral foundations. *Journal of Personality and Social Psychology*, *96*(5), 1029-1046.

Haidt, J., Graham, J., & Joseph, C. (2009). Above and below left–right: Ideological narratives and moral foundations. *Psychological Inquiry*, *20*(2-3), 110-119.

Kline, R. B. (1998). *Principles and practice of structural equation modeling*. New York: Guilford Press.

MacCallum, R. C., Wegener, D. T., Uchino, B. N., & Fabrigar, L. R. (1993). The problem of equivalent models in applications of covariance structure analysis. *Psychological Bulletin*, *114*(1), 185-199.

Raftery, A. E. (1995). Bayesian model selection in social research. *Sociological Methodology*, 111-163.

Sibley, C. G., & Duckitt, J. (2013). The dual process model of ideology and prejudice: A longitudinal test during a global recession. *The Journal of Social Psychology*, *153*(4), 448-466.

Thoemmes, F. (2015) Reversing arrows in mediation models does not distinguish plausible models, *Basic and Applied Social Psychology*, 37(4), 226-234.
