# Supplementary material for: Moral values, social ideologies and threat-based cognition: Implications for intergroup relations
Source: Front Psychol. 2022 Oct 6;13:869121. doi: 10.3389/fpsyg.2022.869121 (PMC9582249; doi:10.3389/fpsyg.2022.869121)
Supplement: Supplementary Table 6 — Models using negative focused attitudes as the outcome variable, Study 2 (N = 388). [file Table_6.docx]

**Appendix**

**Models Using Negative Focused Attitudes as the Outcome Variable, Study 2 (N = 388)**

**Figure A.1**

*Mediation model of Individualizing foundations predicting Negative-Focused Attitudes*

Individualizing

Foundations

SDO

*b* = -.92, *p* < .001

*b* = .69, *p* < .001

Total effect, *b* = -.44, *p* = .001

Direct effect, *b* = .19, *p* = .197

Indirect effect, *b* = -.63, 95% CI [-.90, -.42], *R^2^* = .02, *CSIE* = -.24, *Kappa^2^* = .21

Negative

Attitudes

*Note*. Mediation model of the relationship between individualizing Foundations to negative-focused attitudes by social dominance orientation. All betas represent unstandardized values from the Bootstrap Analysis with 5,000 samples. CSIE represents the Completely Standardized Indirect Effect.

**Figure A.2**

*Mediation model of Binding foundations predicting Negative-Focused Attitudes*

*Note*. Mediation model of the relationship between binding foundations to negative-focused attitudes by right-wing authoritarianism (RWA). All betas represent unstandardized values from the Bootstrap Analysis with 5,000 samples. CSIE represents the Completely Standardized Indirect Effect.

Binding

Foundations

RWA

*b* = 1.00, *p* < .001

*b* = .38, *p* < .001

Total effect, *b* = .65, *p* < .001

Direct effect, *b* = .27, *p* = .055

Indirect effect, *b* = .38, 95% CI [.19, .61], *R^2^* = .10, *CSIE* = .19, *Kappa^2^*= .14

Negative

Attitudes

Following the methods outlined in Study 1, we also produced Structural Equation models to justify the use of the moral foundations as predictor variables for the negative-focused attitudes models reported here (See Supplemental Materials: ‘Alternative Order Analysis’, Figure S.5).

**Threat perceptions, moral foundations, ideologies and negative-focused attitudes**

We again entered threat alongside SDO as a mediator of the individualizing to negative-focused attitudes relationship, and then threat alongside RWA as a mediator of the binding to negative-focused attitudes relationship. We replicated the effects observed for attitudes reported previously; SDO became a non-significant mediator, and RWA became a non-significant mediator when perceived threat was added to the models (See Confidence Intervals for the non-significant indirect effects for Figures A.3 & A.4).

**Figure A.3**

*Mediation model of the relationship between Individualizing Foundations to Negative-Focused Attitudes by Social Dominance Orientation and Threat.*

Individualizing

Foundations

SDO

*b* = -.92, *p* < .001

*b* = .17, *p* = .047

Total effect, *b* = -.44, *p* = .001

Direct effect, *b* = .10, *p* = .438

SDO indirect effect, *b* = -.16, 95% CI [-.38, .04], *CSIE* = -.06

Threat indirect effect, *b* = -.39, 95% CI [-.60, -.23], *CSIE* = -.14

Negative

Attitudes

Threat

*b* = -.56, *p* < .001

*b* = .69, *p* < .001

*Note*. All betas represent unstandardized values from the Bootstrap Analysis with 5,000 samples. CSIE represents the Completely Standardized Indirect Effect.

**Figure A.4**

*Mediation model of the relationship between Binding Foundations to Negative-Focused Attitudes by Right-Wing Authoritarianism (RWA) and Threat.*

Binding Foundations

RWA

*b* = 1.00, *p* < .001

*b* = -.05, *p* = .618

Total effect, *b* = .65, *p* < .001

Direct effect, *b* = .07, *p* = .544

RWA indirect effect, *b* = -.05, 95% CI [-.22, .12], *CSIE* = -.02

Threat indirect effect, *b* = .62, 95% CI [.45, .86], *CSIE* = .31

Negative Attitudes

Threat

*b* = .82, *p* < .001

*b* = .76, *p* < .001

*Note*. All betas represent unstandardized values from the Bootstrap Analysis with 5,000 samples. CSIE represents the Completely Standardized Indirect Effect.
